# A sustainable innovative water remediation approach for methylene blue removal: activated carbon from biomass and synthetic fiber waste

**DOI:** 10.1039/d6ra03333d

**Published:** 2026-08-21

**Authors:** Sarah Magdi, Amina Shaltout, Mahmoud F. Mubarak, Abeer El Shahawy

**Affiliations:** a Department of Civil Engineering, Higher Technological Institute Tenth of Ramadan City 44634 Egypt sara.magdy114@hti.edu.eg; b Department of Civil Engineering, Faculty of Engineering, Suez Canal University P.O. Box 41522 Ismailia Egypt amina_shaltout@eng.suez.edu.eg abeer_shahawi@eng.suez.edu.eg; c Petroleum Applications Department, Egyptian Petroleum Research Institute (EPRI) 1 Ahmed El Zomor St. Nasr City Cairo 11727 Egypt Fathy8753@yahoo.com

## Abstract

The present work focuses on the development of activated carbon materials from two waste sources: natural biomass waste (NBW) and synthetic fiber waste (SFW). This study introduces a novel composite carbon material (CCM) derived from a 75 : 25 mixture of natural waste activated carbon (NWC) and synthetic waste activated carbon (SWC), the first report of such a composite from mixed waste streams. Unlike previous single-precursor studies limited to synthetic solutions, this work systematically optimizes the precursor ratio and validates the CCM on real textile wastewater, achieving 91% removal in synthetic solutions and 75% in real effluent. NBW was activated by sodium hydroxide, and SFW was activated by hydrochloric acid. The prepared materials were characterized by Fourier transform infrared spectroscopy, X-ray diffraction, thermogravimetric analysis, Brunauer–Emmett–Teller (BET), and scanning electron microscopy. Fourier Transform Infrared Spectroscopy analysis showed the presence of oxygen functional groups (O–H, C=O, and C–O), which are responsible for hydrogen bonding and electrostatic interactions with dye molecules. X-ray diffraction confirmed the amorphous nature of the prepared carbons, which provides a large number of adsorption-active sites. BET analysis showed specific surface areas of 465, 280, and 510 m^2^ g^−1^ for NWC, SWC, and CCM, respectively. Batch adsorption experiments were performed to explore the effect of initial dye concentration, contact time, pH, adsorbent dosage, and temperature on the adsorption performance. Under optimized conditions, NWC exhibited 89% removal efficiency with the equilibrium adsorption capacity of 4.45 mg g^−1^ at pH 6, contact time of 10 min, and adsorbent dosage of 0.1 g. SWC showed a removal efficiency of 75% and an equilibrium adsorption capacity of 3.50 mg g^−1^ at pH 7.2, contact time of 50 min, and adsorbent dosage of 0.2 g. CCM (75% NWC + 25% SWC) exhibited the highest removal efficiency of 91% with the equilibrium adsorption capacity of 2.28 mg g^−1^ at pH 7 after 25 min using 0.2 g of adsorbent dosage. As per the Langmuir model, the maximum adsorption capacities for NWC, SWC, and CCM were 13.11, 2.34, and 25.45 mg g^−1^, respectively. The adsorption kinetics of all prepared materials were best described by the pseudo-second-order model (*R*^2^ = 0.998–0.9999), which indicated favorable adsorption kinetics. The equilibrium data of CCM were in good agreement with both Langmuir and Freundlich models, reflecting its mixed microporous and mesoporous structure, which supports both monolayer and multilayer adsorption. Comparing with previously reported adsorbents, CCM showed higher adsorption capacity than date-pit-based adsorbents (18 mg g^−1^) and comparable performance to chitosan/activated carbon composites (22.5 mg g^−1^). Moreover, the optimized CCM was evaluated using real textile wastewater containing high organic loads characterized by chemical oxygen demand (3300 mg L^−1^), biochemical oxygen demand (2000 mg L^−1^), total suspended solids (520 mg L^−1^), and trace inorganic contaminants such as zinc, copper, iron, and chromium. The CCM was found to be effective in dye removal under the complex wastewater conditions with an efficiency of 75%, confirming its practical applicability for sustainable textile wastewater treatment. The dual-waste valorization approach provides an efficient way of transforming problematic waste streams into sustainable adsorbents for wastewater treatment.

## Abstract 2

The present work focuses on the development of activated carbon materials from two waste sources: natural biomass waste (NBW) and synthetic fiber waste (SFW).

## Graphical abstract

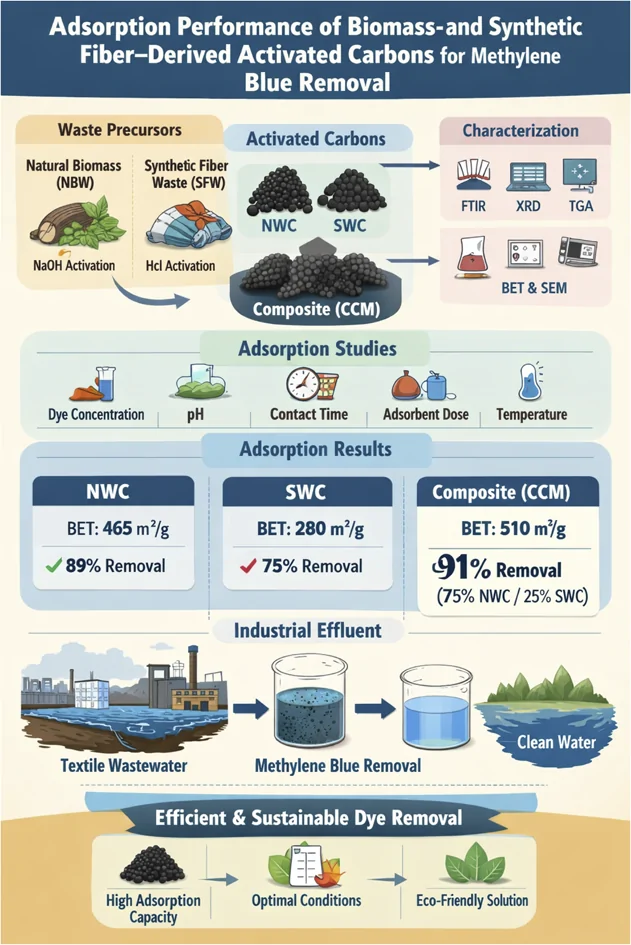

## 1. Introduction

The textile dyeing and printing industries have emerged as important polluters of the world's water bodies, mainly through effluents released by them. Such effluents contain high amounts of chemical oxygen demand (COD), biochemical oxygen demand (BOD), total suspended solids (TSS), and organic pollutants. Methylene blue dye (MB_d_) and other types of artificial dyes are particularly dangerous because of their toxicity, carcinogenicity, and non-biodegradability, which make their removal from aquatic systems extremely difficult.^1,2^ Untreated wastewater loaded with dyes results in reduced light penetration and inhibition of photosynthesis, in addition to bioaccumulation, thus making it a considerable environmental hazard.

Technologies such as oxidation, membrane processes, coagulation, and flocculation, as well as biological techniques, have been widely used in attempts to deal with dye pollutants. However, the limitations of such treatment processes include poor efficiency, secondary sludge formation, high running costs, and a lack of selectivity. Adsorption is an effective and economical process for the elimination of dyes from wastewater due to the variety of inexpensive adsorbents available.^3–5^

Activated carbon (AC) has been referred to as an optimal adsorbent due to its high surface area, excellent porosity, and stability.^6^ Nevertheless, the currently available commercially activated carbon (CAC) has very high costs associated with its production, and therefore, there has been an interest in developing cheaper sources of materials that can be converted to AC. Among such wastes are olive waste, papaya plant biomass, corn cob waste, and waste carbides.^1,4^ However, not only do such methods make wastewater treatment cheaper, but they also play an integral part in waste valorization and the concept of a circular economy.

According to recent research, AC created from biowaste demonstrates more powerful potential for MB_d_ removal than conventional materials due to its heterogeneous structure and multilayer adsorption. In particular, olive-derived AC exhibited a very high dye removal rate for MB_d_ and methyl orange.,^2,7^ while papaya-based carbon gave better performance for MB_d_ and alizarin red S.^4,8^ Similarly, mesoporous carbon obtained from maize also showed high adsorption potential for carcinogenic azo dyes. On the other hand, carbons obtained from industrial wastes, *e.g.*, recycled carbide, have been proven to be efficient in the removal of dyes and COD from real textile wastewater, emphasizing their application in real samples.^1,9^

However, despite these advances, there still exists a major research gap, that is, most adsorption research is still being conducted using synthetic dye solutions. On the other hand, in real wastewaters, different pollutants such as organic materials, heavy metals, and salts compete with the dyes for adsorption, which may decrease the efficiency of the process. In real wastewater, the COD and BOD are usually high, and dissolved solids can inhibit adsorption sites or affect the interaction between the dye and the adsorbent.^1^ This difference shows how important it is to test adsorbents with both artificial and real wastewater to assess whether they can be used.

To overcome these problems, researchers are using composite adsorbents consisting of AC and other materials such as clay, polymers, and other carbons.^10^ These composites are known to work better together, combining the great ability of AC to absorb substances with another material's stability, reusability, and selectivity. An example of the potential of hybrid materials is the clay-carbon composite synthesized from waste bleaching earth that showed higher dye removal efficiency (*E*_r_) than the individual components.^9,10^

Therefore, the aim of this study was to (1) prepare AC from natural biomass waste (NBW) and synthetic fiber waste (SFW) separately and as a composite carbon material (CCM); (2) characterize their structural and surface properties; (3) optimize the adsorption parameters, such as pH (pH_c_), contact time (*t*_c_), adsorbent dosage (Ad_c_), initial concentration (*I*_c_), and temperature (*T*_c_) for MB_d_ removal; (4) evaluate the adsorption kinetics, isotherms, and thermodynamics; and (5) test the adsorbents on real textile wastewater to assess their practical applicability.

While several studies have reported AC production from single waste streams (NBW or SFW) and a few have explored composites, none has systematically optimized the precursor ratio for MB_d_ removal or validated such materials on real textile wastewater with high pollutant loads.^7,9^

To address this gap, the novelty of this work lies in (1) the simultaneous valorization of two distinct waste streams (NBW and SFW); (2) the first systematic optimization of the CCM ratio (75 : 25 natural waste activated carbon (NWC): synthetic waste activated carbon (SWC)), achieving 91% MB_d_ removal; and (3) validation on real textile wastewater, a scenario rarely reported in literature. To the best of our knowledge, this study is the first report of a CCM derived from mixed NBW and SFW precursors with proven performance on real industrial effluent. This dual-waste valorization approach contributes to the circular economy by transforming problematic wastes into a value-added material for water remediation.

## 2. Materials and methods

### 2.1. Chemicals and materials

NBW was collected from local domestic kitchens and consisted of peels of fresh vegetables and fruits such as tomato, potato, lemon, orange, arugula, parsley, pepper, carrot, and cucumber. The peels were rinsed with tap water to remove surface contaminants and diced into small pieces (approx. 1–2 cm). The shredded peel was sun-dried until it was completely dry. The drying time depended on the type of peel. The dried material was used in the activation phase without further milling treatment.

SFW was purchased from Startex Company, 3rd Industrial Zone, 10th of Ramadan City, Egypt. The waste consisted of polyester-based textile fabric scraps, which were cut into square pieces of about 1 × 1 cm. The pieces of cloth were of different colors: pink, purple, black, and white. Before use, the components were cleaned with distilled water to remove surface dust and additives and air-dried in sunlight.

To assess the efficiency of the prepared adsorbents under real conditions, real wastewater samples were collected from the same facility and used for the adsorption experiments. Chemical activation and pretreatment were carried out using analytical-grade sodium hydroxide (NaOH, ≥98% purity, Sigma-Aldrich, USA) and hydrochloric acid (HCl, 37%, Merck, Germany). MB_d_ (C_16_H_18_C_l_N_3_S, MW = 319.85 g mol^−1^, CAS no. 61-73-4) was used as a model cationic dye. Deionized water (DI water) was the working solution.

### 2.2. Preparation for ACs

#### 2.2.1. Preparation of NWC from NBW

**Fig. 1:**
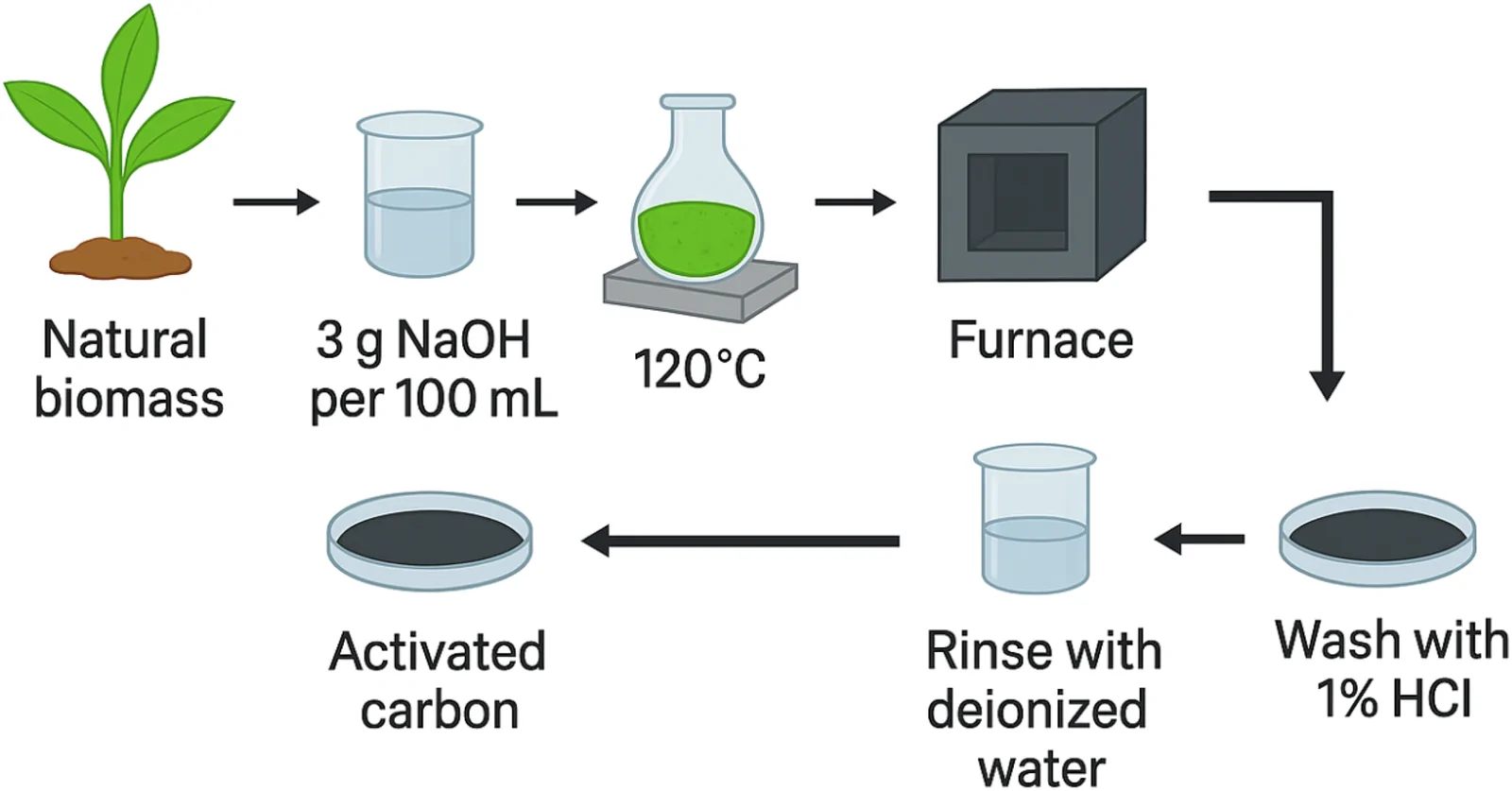
Schematic illustration of the preparation steps of NWC from NBW *via* NaOH activation, pyrolysis at 600 °C for 1.5 h, washing, and drying.

The NBW was first washed with tap water, cut into small pieces (1–2 cm), and air-dried in sunlight until completely dry. For chemical activation, the dried NBW was impregnated with NaOH at an impregnation ratio of 3 : 1 (w/w) (3 g NaOH per 1 g of NBW) in 100 mL of DI water and soaked for 24 h to enhance pore development and to remove impurities. The mixture was then heated on a hot plate for 2 h at 120 °C to intensify the activation process. The impregnated sample was then carbonized at 600 °C in a muffle furnace with a limited air supply (closed crucible) for 1.5 hours. The resultant carbon was cooled to room temperature, ground into a fine powder, and washed repeatedly with DI water and 1% HCl solution until a neutral pH (pH 6–7) was obtained in order to remove excess alkali and mineral impurities. The washed carbon was dried at 105 °C for 12 h and stored for further use. The final yield of NWC was about 28% by weight of the raw NBW. Fig. 1 shows the general preparation procedure of NWC.

The activating agent used for NBW was NaOH due to its known effectiveness on the development of porosity in lignocellulosic materials.^11,12^ NaOH reacts with the cellulose and lignin components, leading to dehydration and cross-linking reactions that produce a highly porous carbon structure.^13^ In addition, NaOH activation has been reported as an effective and cheap route to prepare biomass-derived carbons.^11,12^

#### 2.2.2. Preparation of SWC from SFW

**Fig. 2:**
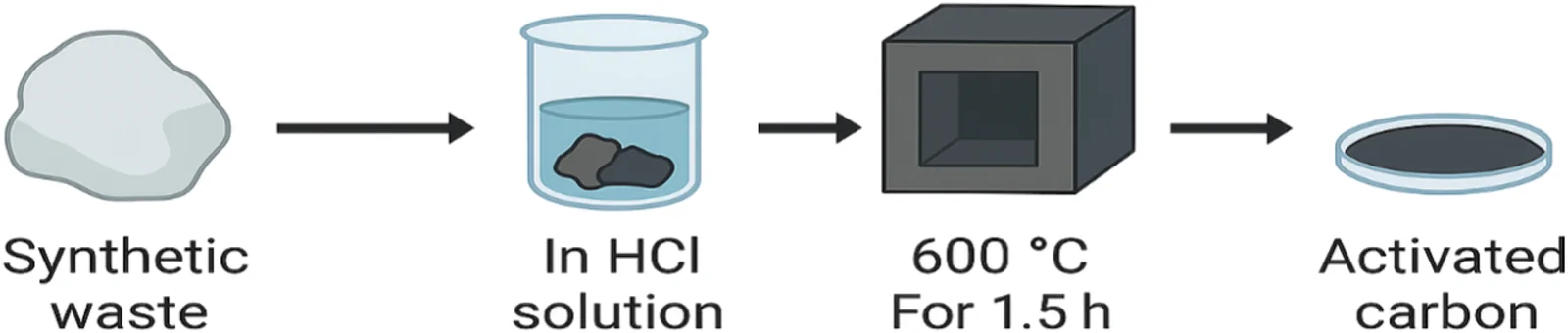
Schematic diagram of preparation steps of SWC from SFW by HCl activation, pyrolysis at 600 °C for 1.5 h, washing, and drying.

The SFW used was supplied by Startex Company (10th of Ramadan City, Egypt) and was made of polyester-based textile fabric off-cuts. The fabric was cut into squares of 1 × 1 cm, washed with distilled water, and air-dried. To remove the residual color and other soluble impurities, the pieces were immersed in a solution of HCl at a volume ratio of 10 mL HCl to 100 mL DI water (10% v/v) for 24 h. Then the impregnated SFW was carbonized at 600 °C for 1.5 h in a muffle furnace under a limited air supply. The resulting SWC was washed with DI water to neutral pH after carbonization, dried at 80 °C for 6 h, and ground. The final SWC yield was approximately 32% w/w of the raw SFW. The preparation process of SWC is summarized in Fig. 2.

HCl was selected for SFW because it could remove inorganic impurities and open the pore structure of textile fibers.^14,15^ HCl treatment is effective in removing metal oxides and other mineral contaminants that may block the pores and hence improve the accessibility of active sites.^16^ This approach has been previously reported to activate carbon fibers derived from textile waste.^9^

#### 2.2.3. Preparation of CCM

The prepared NWC and SWC were physically mixed in different weight ratios (25 : 75, 50 : 50, and 75 : 25) for preparing the CCM. The mixture was well homogenized in a mortar and pestle and used as the composite adsorbent without further thermal treatment. The best result was observed at a ratio of 75% NWC and 25% SWC.

### 2.3. Experimental procedures

NWC, SWC, and CCM were the three adsorbents used in batch adsorption studies to assess the MB_d_ adsorption performance. The effects of different parameters like *T*_c_ (30–60 °C), Ad_c_, *I*_c_, *t*_c_ (0–90 min), and pH_c_ (4–11) on *E*_r_ were explored. In each experiment, 50 mL of MB_d_ solution with *I*_c_ (10–30 mg L^−1^) was mixed with Ad_c_ (0.1–0.25 g). For the chosen contact period at room temperature, the flasks were shaken at a constant speed of 100 rpm, while the pH_c_ of the solutions was adjusted using either 0.1 M HCl or 0.1 M NaOH. After agitation, the solutions were filtered. A UV-Vis spectrophotometer (Specord 210 plus, Analytik Jena, Germany) set to 665 nm was used to measure the residual concentration of MB_d_. All batch adsorption experiments were performed in triplicate (*n* = 3), and the averages are reported as mean ± standard deviation (SD). The error bars in the figures represent the SD of three independent measurements. The equilibrium adsorption capacity (*C*_qe_) and *E*_r_ were determined from the *I*_c_ and the equilibrium concentrations (*E*_c_) using the standard equations. The optimization was performed using the one-factor-at-a-time (OFAT) method, where one parameter was varied, and the values of the rest of the parameters were kept at their initial values. The ranges studied were *I*_c_ (10–30 mg L^−1^), *t*_c_ (0–90 min), pH_c_ (4–11), Ad_c_ (0.05–0.25 g), and *T*_c_ (30–60 °C). The best value of each parameter was the one that showed the highest *E*_r_.

### 2.4. Kinetics, isotherm, and thermodynamics adsorption experiments

Batch adsorption experiments were performed to investigate the *E*_r_ of MB_d_ using prepared ACs samples and different operating conditions, *i.e.*, *t*_c_, *I*_c_, pH_c_, *T*_c_, and Ad_c_. These procedures are reported in recent adsorption studies.^17,18^*E*_r_ and *C*_qe_ were used to characterize the adsorption performance.^19^ The *E*_r_ was calculated using eqn (1):1
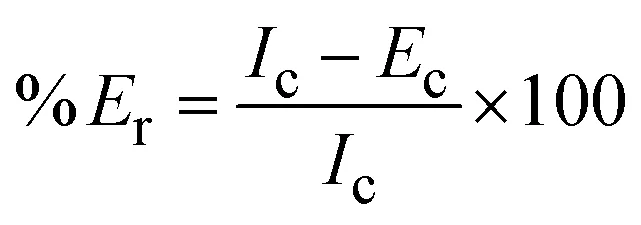
where *I*_c_ and *E*_c_ (mg L^−1^) reflect the concentrations of MB_d_.^20^

The *C*_qe_ (mg g^−1^) was calculated using eqn (2):2
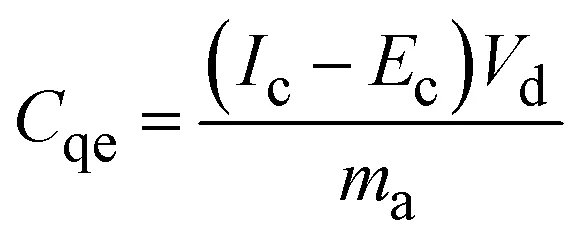
where *V*_d_ (L) is the volume of the dye solution and *m*_a_ (g) is the mass of the adsorbent.^21^

The experimental data were fitted to the pseudo-first-order model (PFO_m_) and pseudo-second-order model (PSO_m_) to study adsorption kinetics and determine the adsorption rate and possible governing mechanisms, which still remain the most commonly used kinetic models in current dye adsorption studies.^22,23^ The PFO_m_ is expressed as eqn (3):

3 ln(*C*_qe_ − *C*_qt_) = ln(*C*_qe_) − *k*_1_·*t*_m_

While the PSO_m_ was expressed as eqn (4):4
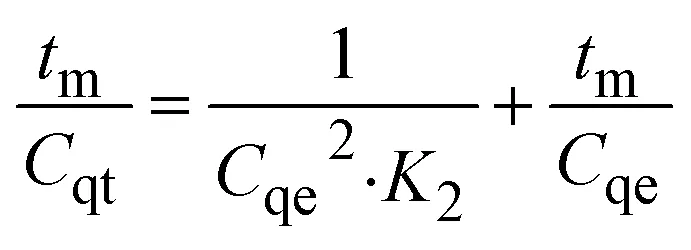
where *C*_qt_ (mg g^−1^) is the adsorption capacity at time (*t*_m_), respectively; *k*_1_ (min^−1^) is the PFO_m_ rate constant; and *k*_2_ (g mg^−1^ min^−1^) is the PSO_m_ rate constant.

The MB_d_ adsorption behavior onto the produced adsorbents was described using the Langmuir and the Freundlich models for equilibrium isotherm analysis.^11,23^Eqn (5) represents the Langmuir isotherm model, which assumes monolayer adsorption on a homogeneous surface.5
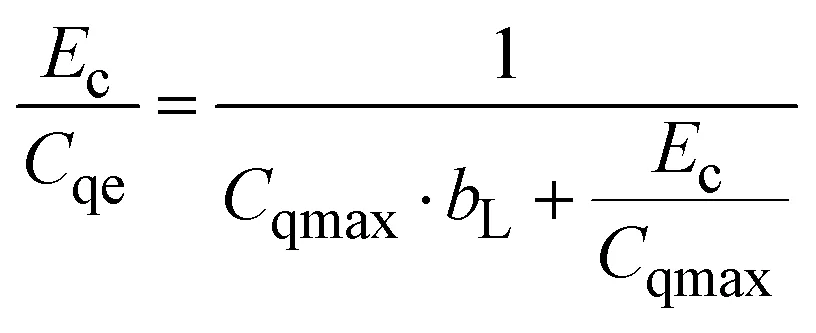
where *b*_L_ (L mg^−1^) is the Langmuir constant and *C*_qmax_ (mg g^−1^) is the maximum adsorption capacity.

The Freundlich isotherm (eqn (6)) describes multilayer adsorption on a heterogeneous surface.6
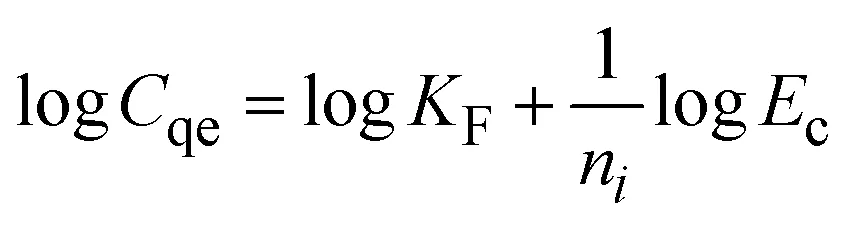
where *K*_F_ (mg g^−1^)(L mg^−1^)^1/*n*_*i*_^ is the Freundlich constant corresponding to *C*_qmax_ and *n*_*i*_ is the adsorption intensity. The goodness of fit for each model was checked by the correlation coefficient (*R*^2^).^24^

The thermodynamic parameters were investigated to evaluate the nature of MB_d_ adsorption on NWC, SWC, and CCM. Batch adsorption experiments were performed at different *T*_c_ to study the effect of *T*_c_ on *E*_r_.

To assess the nature and viability of MB_d_ adsorption onto NWC, SWC, and CCM, thermodynamic parameters were examined. Batch adsorption experiments were performed at different *T*_c_ to investigate the effect of *T*_c_ on *E*_r_.^25,26^ The standard Gibbs free energy change (Δ*G*_s_) (J mol^−1^) was calculated using eqn (7):

7 Δ*G*_*s*_ = −*R*·ln(*K*_c_)

where *T*_k_ is the absolute temperature (K), *K*_c_ is the equilibrium constant, and *R* is the universal gas constant (8.314 J mol^−1^ K^−1^).^13^ The Van't Hoff eqn (8) was used to calculate the standard enthalpy change (Δ*H*_s_) and entropy change (Δ*S*_s_):8
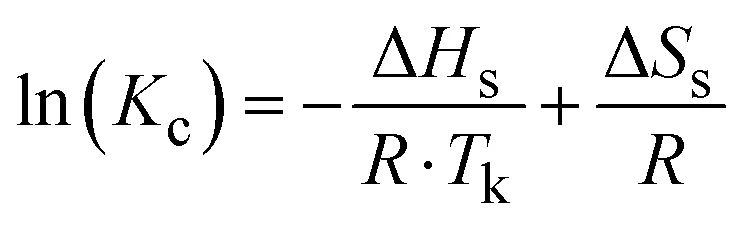


The plotting of ln(*K*_c_) *versus T*^−1^ yields Δ*H*_s_ (J mol^−1^) from the slope and Δ*S*_s_ (J mol^−1^ K^−1^) from the intercept. These thermodynamic characteristics reveal the degree of disorder, heat changes, and spontaneity associated with the adsorption mechanism.^21^

### 2.5. Material characterization

The prepared ACs (NWC and SWC) were characterized by various techniques. The surface morphology, pore distribution, and particle shape were observed by scanning electron microscopy (SEM).^27,28^ The nitrogen adsorption–desorption isotherms were used to determine the specific surface area, pore volume, and pore size distribution by the Brunauer–Emmett–Teller (BET) method.^12^ Thermal stability was studied by thermogravimetric analysis (TGA) under a nitrogen atmosphere.^29^ Fourier Transform Infrared Spectroscopy (FTIR) in the range of 400–4000 cm^−1^ was used to identify surface functional groups (hydroxyl, carboxyl, and carbonyl).^14,30^ Finally, the crystalline structure and amorphous carbon content were analyzed by X-ray diffraction (XRD) with Cu-Kα radiation.^31^

### 2.6. Identifying the zero charge point (pH_zcp_)

The pH drift method was used to find the point of zero charge pH (pH_zcp_) of the prepared AC samples, which is also used in recent studies.^32,33^ The initial pH_c_ (pH_ci_) of a 50 mL NaCl solution (0.01 M) was tuned to a range of values between 2 and 9 with 0.1 M HCl or NaOH. Then, a constant amount of the adsorbent (0.10 g) was added to each flask, and the suspensions were shaken for the predetermined time of equilibration. After the settling of the suspensions, the final pH_c_ (pH_cf_) of each solution was recorded, and the difference between the pH_ci_ and pH_cf_ values, ΔpH_c_, was calculated.

### 2.7. Real textile wastewater characterization

Real textile wastewater was obtained from Startex Company, 10th of Ramadan City, Egypt. The sample was collected from the main discharge point of the dyeing unit during the normal production hours and preserved in sterile polyethylene containers and immediately transported to the laboratory. The sample was homogenized and filtered through Whatman no. 1 filter paper to remove suspended particles before analysis.

The effluent was analyzed for its physicochemical parameters by standard methods. pH was measured using a calibrated digital pH meter, and electrical conductivity (EC) and total dissolved solids (TDS) were measured using a conductivity meter. The *I*_c_ of dye was measured at 665 nm by a UV-Vis spectrophotometer and was found to be around 80 mg L^−1^ (equal to 80 mg per L MB_d_ equivalent). COD was determined using the standard dichromate oxidation method and BOD_5_ by the 5-day incubation method. TSS was measured by the gravimetric method. Heavy metals such as zinc (Zn), copper (Cu), iron (Fe), and chromium (Cr) were determined by atomic absorption spectrophotometry (AAS) using a Shimadzu UV-1800 (Japan). The color intensity was measured as absorbance at 665 nm using a UV-Vis spectrophotometer. The results are shown in Table 1.

**Table 1:** Physicochemical properties of the raw industrial wastewater sample that was obtained from Startex Company (10th of Ramadan City, Egypt)

Parameter	Value	Unit	Method
pH	9.5	—	Digital pH meter
EC	1000	µS cm^−1^	Conductivity meter
TDS	650	mg L^−1^	Gravimetric
*I*_c_ of dye	80	mg L^−1^	UV-vis at 665
COD	3300	mg L^−1^	Dichromate oxidation method
BOD_5_	2000	mg L^−1^	5 day incubation method
TSS	520	mg L^−1^	Gravimetric analysis
Color (absorbance at 665 nm)	0.85	—	UV-vis spectrophotometer
Zn	0.1	mg L^−1^	AAS
Cu	0.05	mg L^−1^	AAS
Fe	0.3	mg L^−1^	AAS
Cr	0.02	mg L^−1^	AAS

## 3. Results and discussion

### 3.1. Material characterization

#### 3.1.1. SEM analysis

**Fig. 3:**
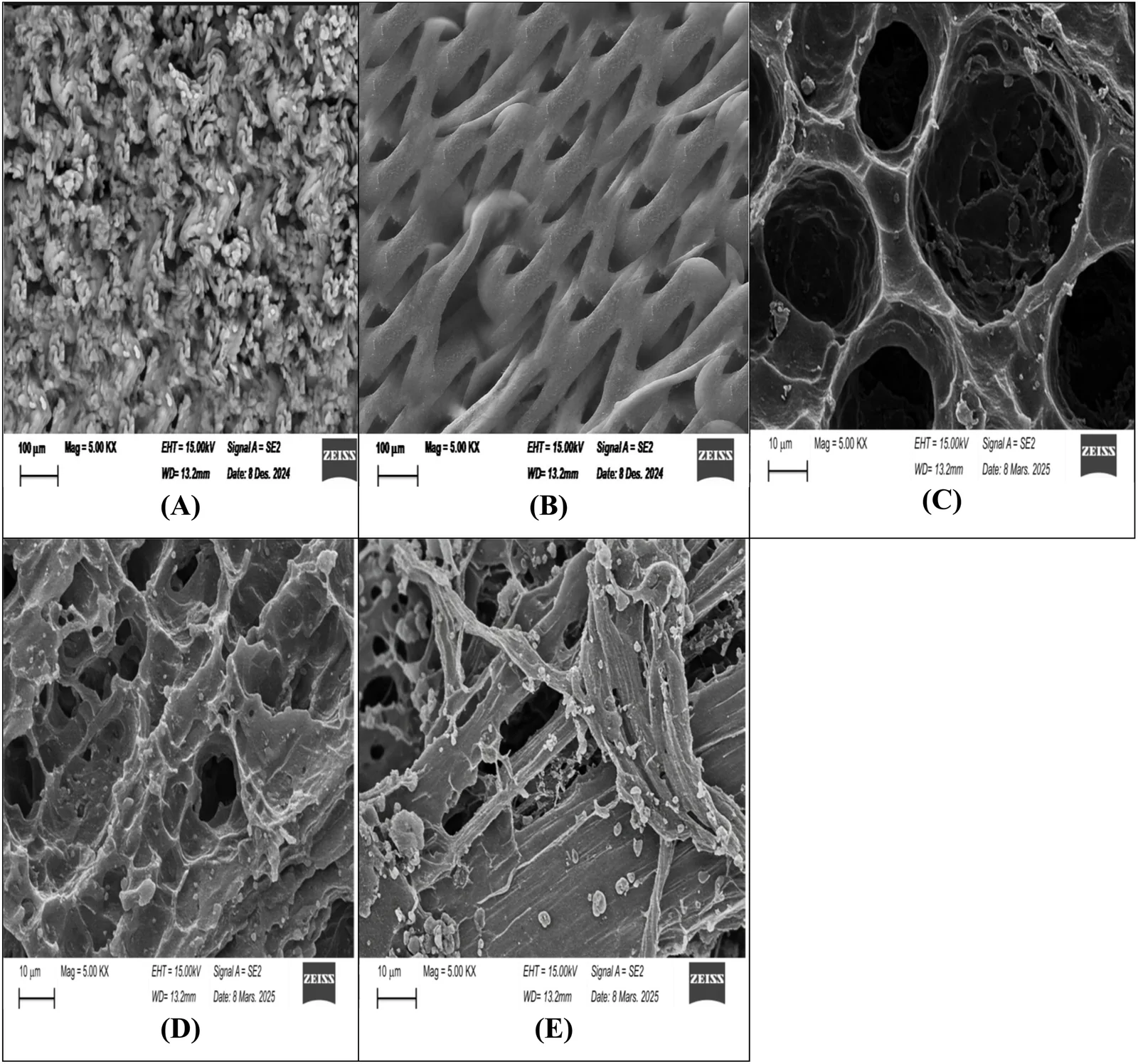
SEM micrographs showing the surface morphology: (A) NBW, (B) SFW, (C) NWC, (D) SWC, (E) CCM.

The SEM micrographs, presented in Fig. 3, completely show the morphological evolution of the materials from raw materials to ACs. The rough and irregular morphology of NBW with inherent cavities and a heterogeneous surface indicates a strong potential for pore formation.^13^ Following NaOH activation, NWC exhibits a highly porous structure with many small to medium-sized pores uniformly distributed on its surface and thin pore walls. The increase in porosity is related to the etching action of NaOH, which significantly enhances the surface roughness and surface area, thus confirming the effectiveness of alkaline activation for the fabrication of high-quality adsorbents from biomass.^11,12,28^ Also, recent studies have confirmed that NaOH activation improves the surface area and pore connectivity of biomass-derived carbons.^34^

By contrast, SFW shows a smoother and more compact morphology with limited porosity and a few cracks, which indicates a lower tendency to spontaneous pore formation. After activation with HCl, SWC presents a relatively smooth surface with fewer and smaller cavities, which explains its lower adsorption performance compared to NWC.^14^ The CCM structure is a mixture of the irregular pores of NWC with the denser and more stable surface of SWC, as shown in Fig. 3. This hybrid morphology leads to a highly porous structure similar to NWC, but with the structural stability of SWC; thus, it is more effective for adsorption than either individual material.^13,30^

SEM observations of the morphological changes confirm the successful activation of both precursors. The formation of pores in NWC and CCM promotes fast diffusion of the dye molecules to the active sites, whereas the low porosity of SWC is associated with slow kinetics of dye adsorption and lower *E*_r_. As shown in Fig. 3.^11,12^ The better performance of CCM (91% *E*_r_) can be ascribed to the balanced pore structure, which is a combination of the high porosity of NWC and the mechanical stability of SWC.

#### 3.1.2. FTIR analysis

**Fig. 4:**
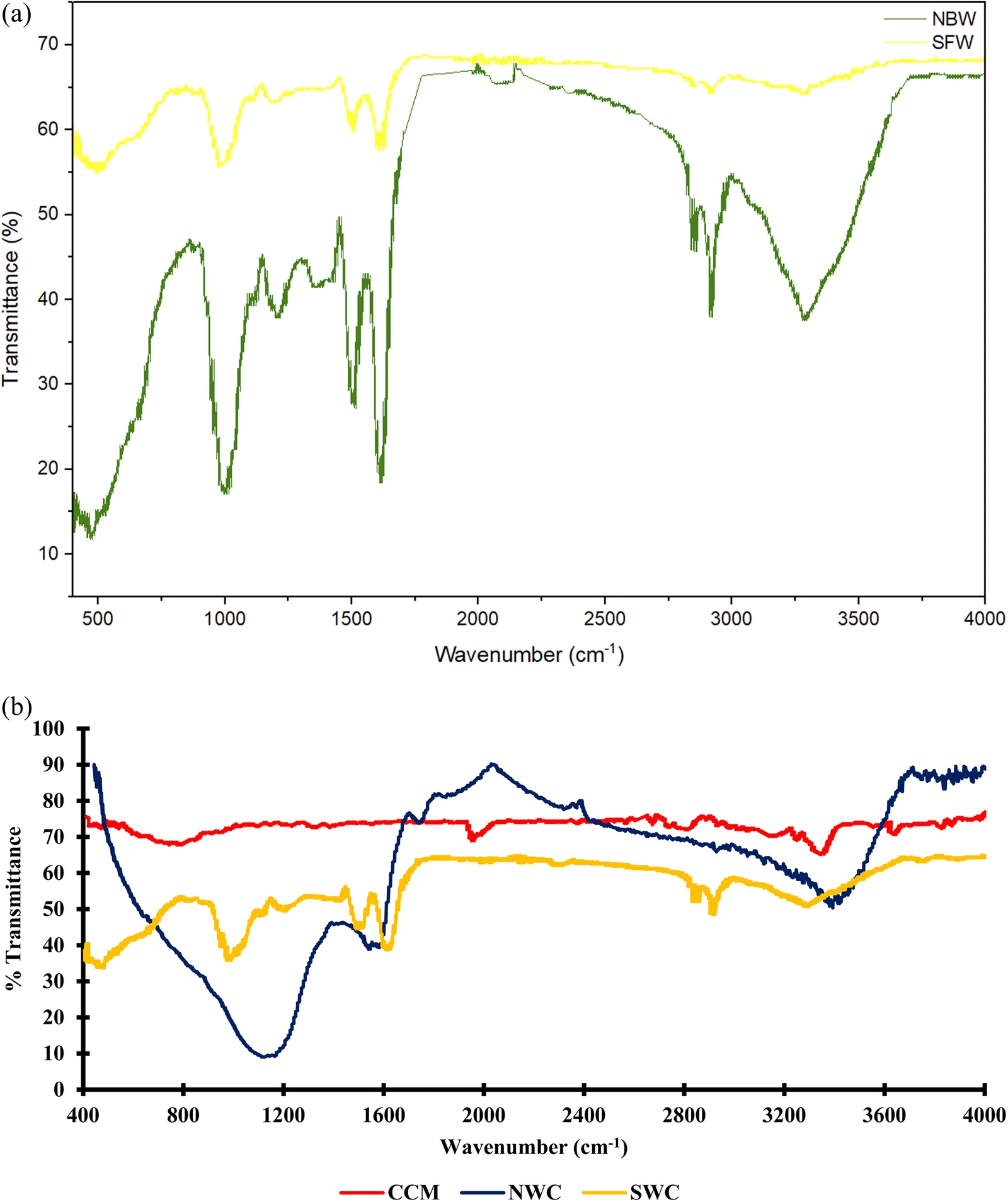
(a) FTIR spectra of the raw materials: NBW and SFW. (b) FTIR spectra of the ACs: NWC, SWC, and CCM.

Fig. 4a FTIR spectra of raw materials (NBW and SFW). Fig. 4b FTIR spectra of ACs (NWC, SWC, and CCM). The NBW spectrum has distinct peaks at around 3400 cm^−1^ (O–H stretching), 2920 cm^−1^ (C–H stretching), 1730 cm^−1^ (C=O stretching from lignin and hemicellulose), and 1000–1200 cm^−1^ (C–O stretching in polysaccharides). The NWC spectrum after activation shows a decrease or shift of these peaks, especially the one around 1730 cm^−1^ related to the degradation of cellulose, hemicellulose, and lignin, and the formation of new aromatic carbonaceous structures, as shown in Fig. 4b. The raw fibers present characteristic bands of polyester in the SFW spectrum. The SWC spectrum after activation shows peak broadening and a significant decrease in the intensity of the O–H and C=O bands, indicating a partial elimination and modification of functional groups.^15,16^ The CCM spectrum (also shown in Fig. 4b) exhibits signals in the range of ∼1000–1200 cm^−1^ (the C–O stretching), a band at ∼1600 cm^−1^ (the C=C stretching in aromatic structures), and a very strong peak near ∼3400 cm^−1^ (the O–H stretching), an indication of the presence of oxygen-containing groups derived from both precursors.^35^ These spectral changes confirm the chemical activation effectively modifies the surface chemistry by reducing the amount of oxygenated functional groups, increasing aromaticity, and improving the adsorptive properties of the carbons.

The FTIR results also give some insight into the adsorption mechanism. The oxygen-containing functional groups (O–H, C=O, C–O) remaining on the surfaces of NWC and CCM are crucial for MB_d_ adsorption, as they can create hydrogen bonds with the nitrogen atoms of the dye molecules, as shown in Fig. 4a and b. Furthermore, such groups increase the electrostatic attraction between the negatively charged carbon surface and the positively charged MB_d_^+^ cations.^13,27^ The disappearance or fading of these peaks after activation is a confirmation of the successful conversion of the starting materials into carbonaceous structures with altered surface chemistry. The better performance of CCM (91% *E*_r_) than SWC (75%) can be partly attributed to the retention of oxygen-containing functional groups from the NWC fraction, along with the stable carbon skeleton of the SWC, as clearly observed in the CCM spectrum in Fig. 4b.

#### 3.1.3. BET surface area and pore structure analysis

**Fig. 5:**
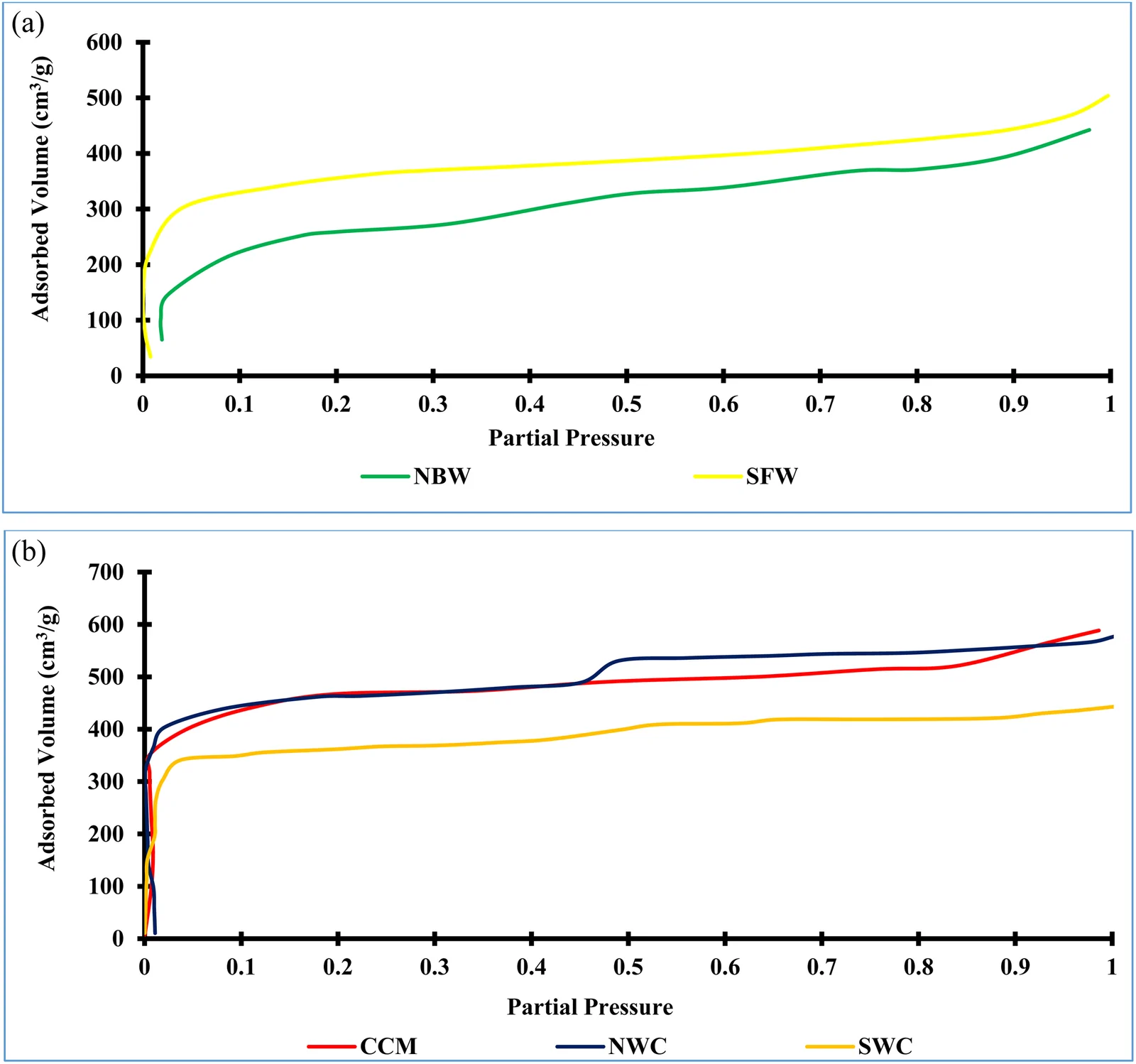
(a) BET nitrogen adsorption–desorption isotherms of the raw materials: NBW and SFW. (b) BET nitrogen adsorption–desorption isotherms of the ACs: NWC, SWC, and CCM.

The BET nitrogen adsorption–desorption isotherms of the raw materials (NBW and SFW) and the ACs (NWC, SWC, and CCM) are presented in Fig. 5a and b. The NBW isotherm shows very low adsorption, while NWC shows a much higher volume of adsorption over the entire pressure range, confirming the increase of porosity and surface area during activation.^36^ The SFW and SWC curves show the expected shape for Type I isotherms, which are typically found for microporous materials. The adsorption volume of SWC is slightly greater at higher relative pressure, which suggests the development of pores after activation.^37^ The CCM isotherm exhibits a steep rise at low relative pressure and a gradual rise, suggesting the contribution of both micropores and mesopores to the overall adsorption characteristics.^29^

The large enhancement of specific surface area from raw materials to ACs shows the efficiency of the activation process. The surface area of CCM (510 m^2^ g^−1^) with the pore volume (0.487 cm^3^ g^−1^) and average pore diameter (3.82 nm) as shown in Table 2 are superior to those of NWC (465 m^2^ g^−1^, 0.412 cm^3^ g^−1^, 3.55 nm) and SWC (280 m^2^ g^−1^, 0.215 cm^3^ g^−1^, 3.07 nm). Furthermore, CCM exhibits a more favorable distribution of micropore and mesopore surface area (325 and 185 m^2^ g^−1^, respectively), indicating its greater potential in adsorption applications. The excellent *E*_r_ (91%) of CCM was directly attributed to its high surface area, which provides more available active sites for dye molecules.^38^ The BET isotherms obtained show that chemical activation is a suitable method to enhance specific surface area and pore volume, particularly for CCM, thereby increasing their efficiency in adsorption applications.

**Table 2:** BET surface area and pore structure parameters of the prepared carbon materials

Parameter	NWC	SWC	CCM
BET surface area (m^2^ g^−1^)	465	280	510
Pore volume (cm^3^ g^−1^)	0.412	0.215	0.487
Average pore diameter (nm)	3.55	3.07	3.82
Micropore surface area (m^2^ g^−1^)	290	160	325
Mesopore surface area (m^2^ g^−1^)	175	120	185

#### 3.1.4. Distribution analysis of pore size (DAPZ)

**Fig. 6:**
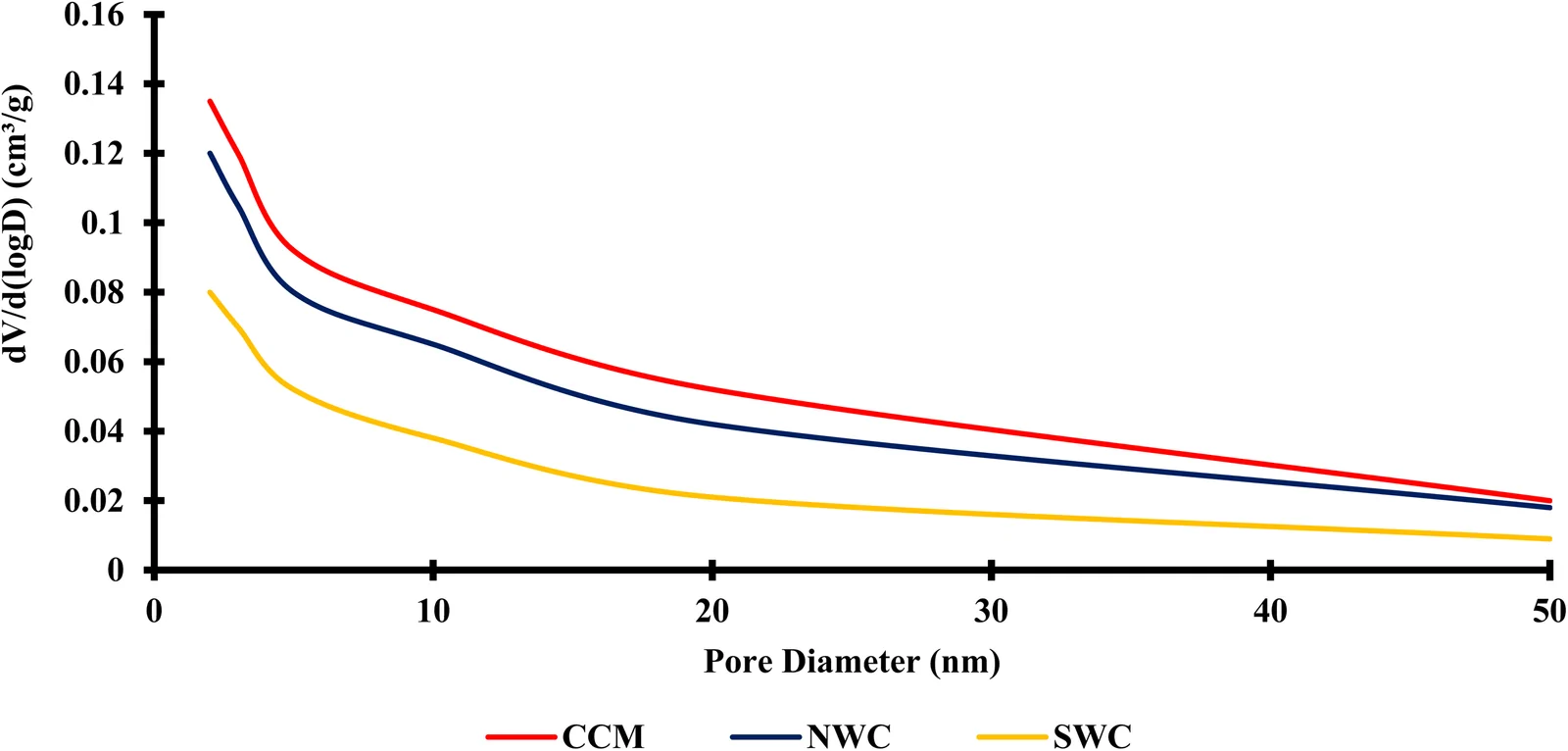
DAPZ of the prepared carbon materials (NWC, SWC, and CCM).

The pore size distribution obtained from DAPZ analysis showed significant differences between the samples studied. The NWC showed moderate pore volume and relatively good porosity, but lower than the CCM. The SWC had the lowest pore volume in the whole range of pore diameters, which indicates its relatively low *C*_qmax_. On the other hand, the CCM had the highest differential pore volume across the entire range of pore diameters; this is a result of the synergistic effect achieved when NWC and SWC are combined to form the CCM. This result is also representative of a favorable balance between micro- and mesopore volumes in the CCM material, which is highly desirable in adsorption applications due to the potential for the adsorption of both small and large adsorbate molecules. This data further confirms that a CCM can be used to improve the pore structure and increase the *E*_r_. Fig. 6 shows the results obtained.

#### 3.1.5. XRD analysis

**Fig. 7:**
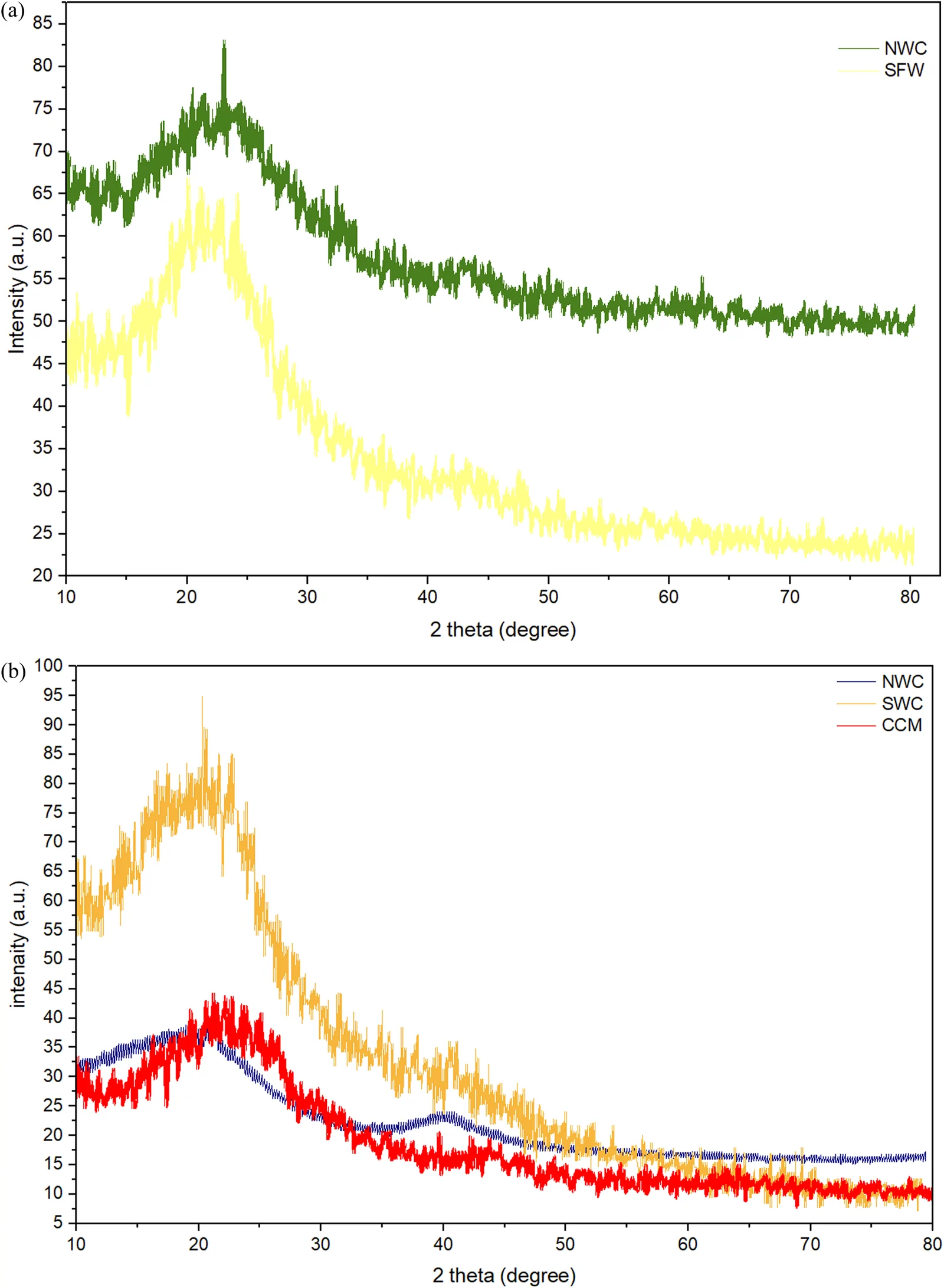
(a) XRD patterns of the raw materials: NBW and SFW. (b) XRD patterns of the ACs: NWC, SWC, and CCM.

The XRD patterns of the raw materials (NBW and SFW) are shown in Fig. 7a and the ACs (NWC, SWC, and CCM) in Fig. 7b. The XRD pattern of NBW shows a broad peak around 2*θ* = 20–30°, which is attributed to the semi-crystalline structure because of the presence of cellulose and lignin.^39^ NWC shows a decreased intensity and a wider peak on activation, indicating the conversion to a more amorphous structure with better porosity.^9^ Conversely, SFW raw fibers exhibit sharp crystalline peaks at around 2*θ* = 22–25°, which correspond to the ordered arrangement of polymeric textile fibers. However, after carbonization and activation, the sharp peaks disappear, and SWC exhibits a predominantly amorphous halo owing to the structural degradation of the crystalline regions and the formation of disordered carbon structures with good adsorption capabilities.^15^ The CCM pattern is presented in Fig. 7b, with one broad peak at 2*θ* = 23–26°, characteristic of a predominantly amorphous structure with properties of both NWC and SWC.^16^ As shown in Fig. 7b,^40^ the thermal treatment was efficient to produce highly disordered carbon structures; no crystalline peaks could be observed for any of the ACs.

The prepared ACs are amorphous in nature, which has been confirmed by XRD results showing broad peaks in the region of 2*θ* = 20–30°. This amorphous character is advantageous for adsorption because it results in a disordered carbon matrix with a large number of active sites and accessible pore networks.^33,40^ The absence of sharp crystalline peaks in NWC, SWC, and CCM shows that the thermal treatment was successful in converting the raw materials to carbon with turbostratic structure, which is characteristic of the ACs with high surface area and porosity.^16^ This is due to the wider peak of CCM, which indicates more disorder, which might be related to its better adsorption ability (91% *E*_r_) by providing more adsorption sites than NWC (89%) and SWC (75%), as is clearly shown in Fig. 7b.

#### 3.1.6. TGA analysis

**Fig. 8:**
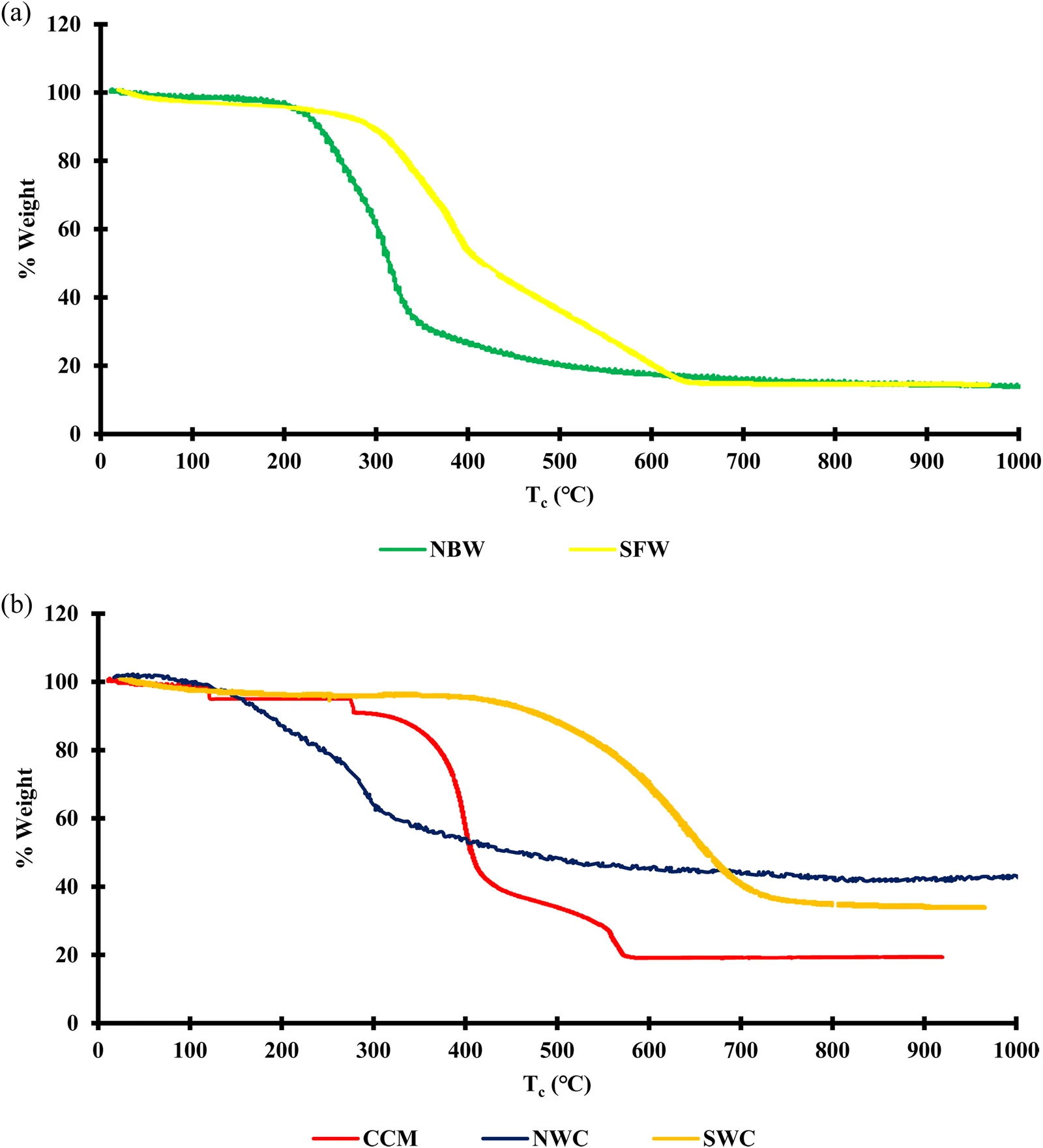
(a) TGA thermograms of the raw materials: NBW and SFW. (b) TGA thermograms of the ACs: NWC, SWC, and CCM.

The TGA thermograms of the raw materials (NBW and SFW) are shown in Fig. 8a, and the thermograms of the ACs (NWC, SWC, and CCM) are shown in Fig. 8b. NBW loses weight below 150 °C as moisture evaporates. The degradation of hemicellulose, cellulose, and lignin is important between 200 and 400 °C.^41^ The activation of NWC leads to lower mass loss and higher residual carbon, which indicates improved thermal stability. This phenomenon is apparent from Fig. 8b.^42^ SFW loses moisture from the raw fibers at low temperatures and shows a major decomposition step between 250 and 500 °C due to the breakdown of polymers, while SWC shows improved stability and higher residual weight after activation.^24^ CCM, Fig. 8b, shows one major weight loss step between 200 and 500 °C, and the weight remains almost constant thereafter, confirming the formation of thermally stable, carbon-rich material.^42^

The increased thermal stability of the ACs compared to the raw materials proves that the carbonization and activation process was performed successfully, as shown in Fig. 8a and b. The increased residual mass of NWC, SWC, and CCM reveals the formation of stable carbon structures. The stability of CCM is particularly critical for practical application where thermal durability is required. All the carbons show an increase in thermal stability due to the activation process. The most stable performance is observed for CCM in general.

### 3.2. The impact of different variables on the ACs

#### 3.2.1. Effect of pH_c_

**Fig. 9:**
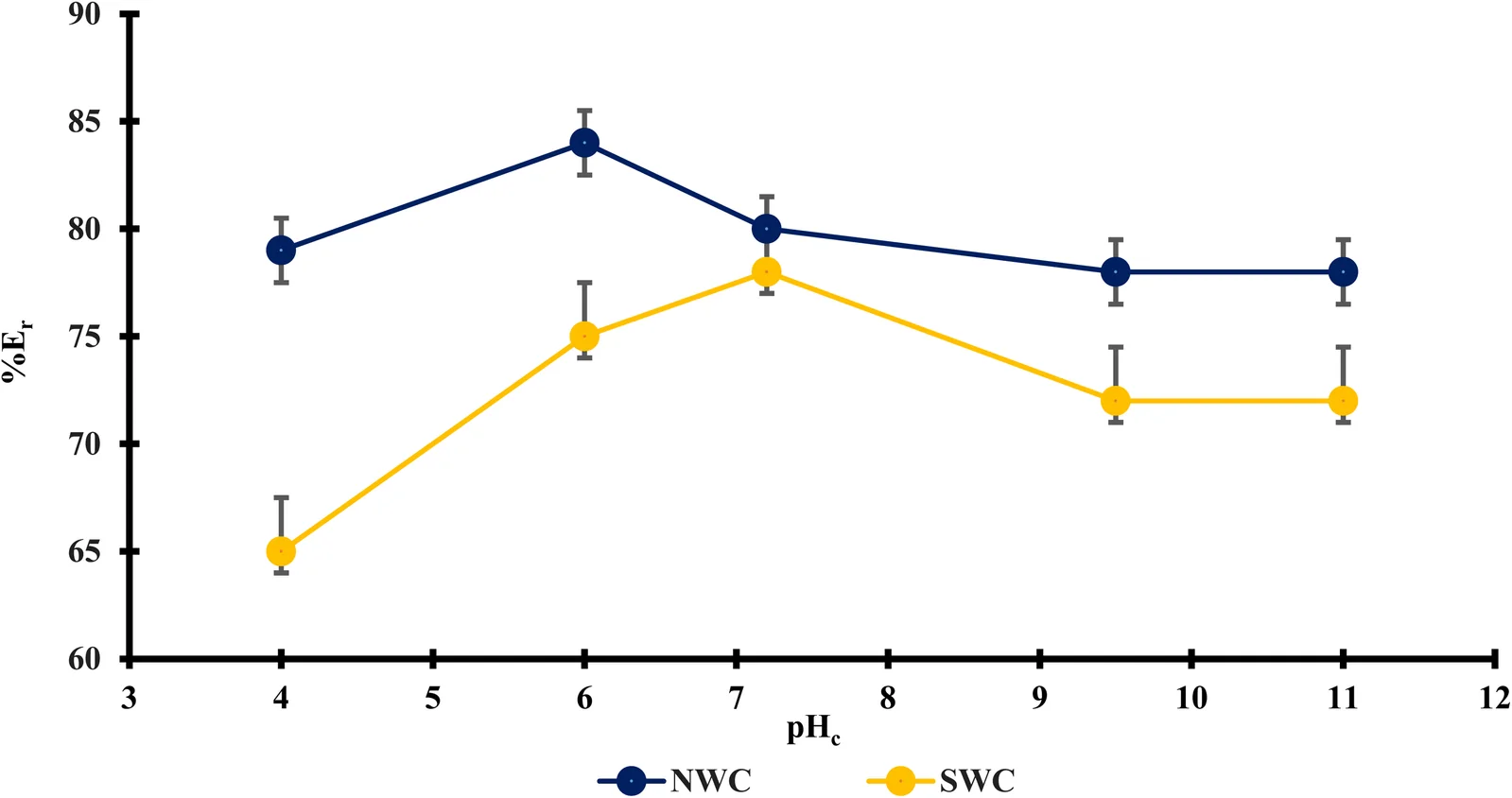
Effect of solution pH_c_ on the *E*_r_ of MB_d_ by NWC and SWC. Error bars represent SD (*n* = 3).

As shown in Fig. 9, the optimum pH_c_ for maximum dye removal using NWC was 6, achieving 89% *E*_r_ with *C*_qe_ = 4.45 mg g^−1^, whereas SWC showed slightly higher performance at pH_c_ 7.2, achieving 75% *E*_r_ with *C*_qe_ = 3.50 mg g^−1^ (summarized in Table 3 along with CCM performance). Adsorption decreases at lower pH_c_ levels because the carbon surface becomes protonated, leading to electrostatic repulsion with cationic dye molecules. In almost neutral and slightly basic conditions, losing protons increases the negative charges on the surface, which helps absorb more dye.^43^ However, the ability to hold onto the dye remained steady within the tested pH_c_ range, which further confirmed the strong dye removal ability of both carbons in different conditions.^44^

**Table 3:** Optimal conditions for MB_d_ adsorption onto NWC, SWC, and CCM

Parameters	NWC	SWC	CCM
Optimal pH_c_	6	7.2	7
Optimal *t*_c_ (min)	10	50	25
Optimal Ad_c_ (g)	0.1	0.2	0.2
Optimal *I*_c_ (mg L^−1^)	10	10	10
Optimal *T*_c_ (°C)	30	30	30
*E*_r_ (%)	89	75	91
*C*_qe_ (mg g^−1^)	4.45	3.50	2.28
*C*_qmax_ (mg g^−1^)	13.11	2.34	25.45

#### 3.2.2. Effect of Ad_c_

**Fig. 10:**
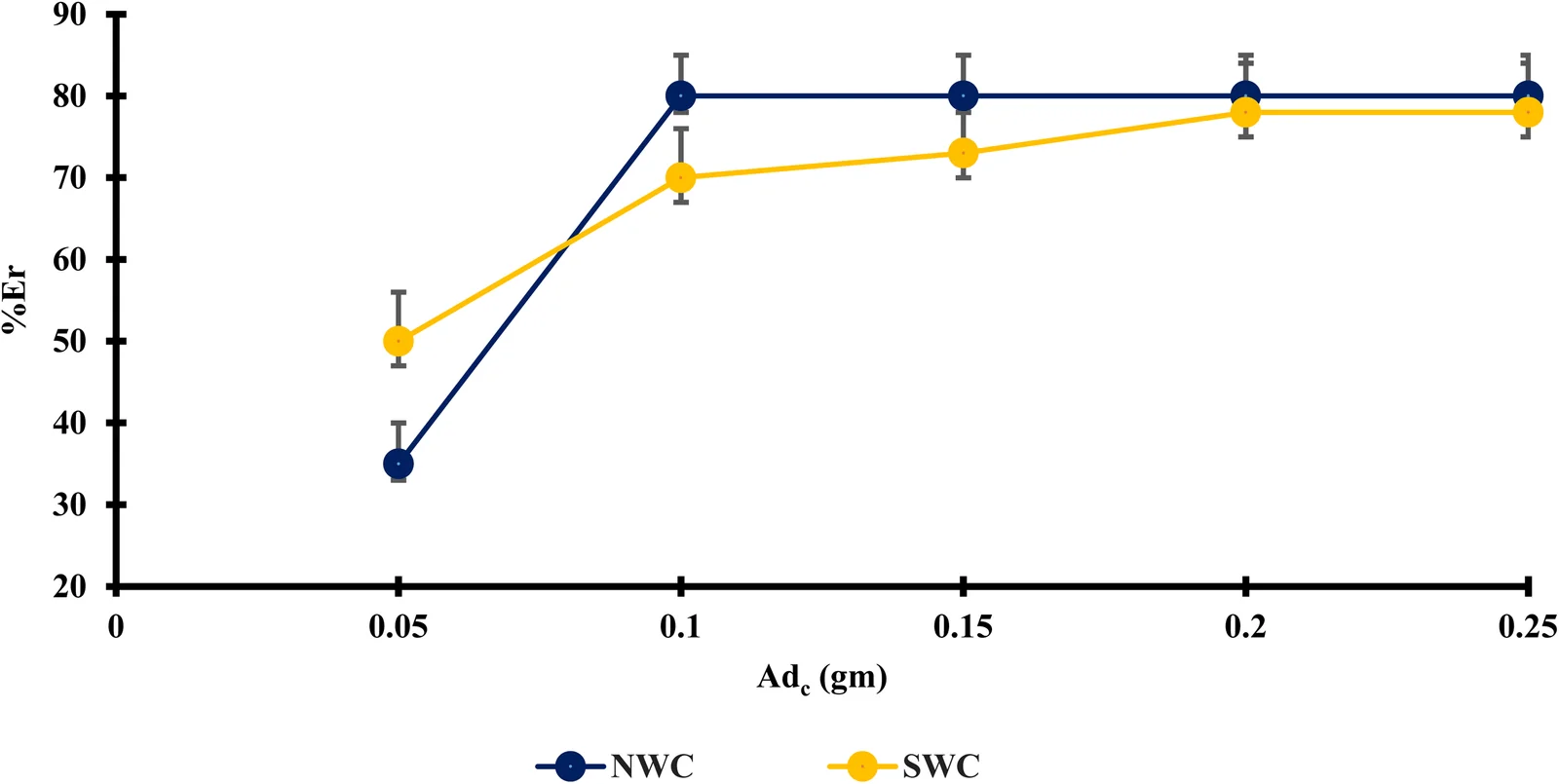
Effect of Ad_c_ on the % *E*_r_ of MB_d_ by NWC and SWC. Error bars represent SD (*n* = 3).

Fig. 10 illustrates the effect of Ad_c_ on the *E*_r_ of MB_d_ using NWC and SWC. Due to the higher number of active sites available on NWC, *E*_r_ increased as Ad_c_ increased from 0.1 to 0.25 g; however, the optimal Ad_c_ was 0.1 g, achieving 89% *E*_r_ with a *C*_qe_ of 4.45 mg g^−1^. This demonstrates that NWC can provide sufficient active sites even at lower Ad_c_ because of its larger surface area. In contrast, SWC required a higher optimal Ad_c_ of 0.2 g to reach 75% *E*_r_ with a *C*_qe_ of 3.50 mg g^−1^, due to its denser pore structure that requires a higher Ad_c_ to provide enough surface area.^45^

**Fig. 11:**
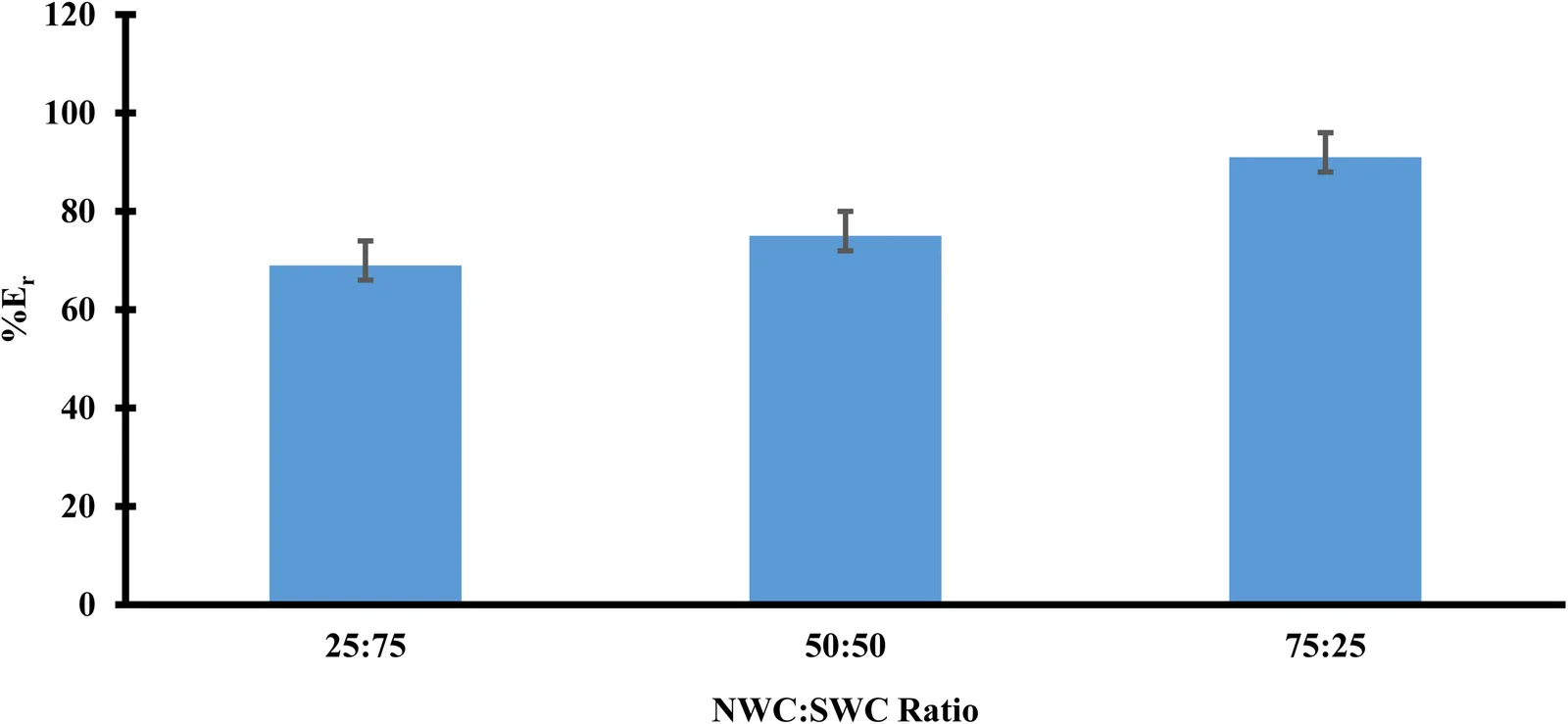
% *E*_r_ of MB_d_ at different NWC : SWC ratios. Error bars represent SD (*n* = 3).

Fig. 11 shows the performance of CCM at a constant Ad_c_ of 0.2 g with different ratios of NWC : SWC (25 : 75, 50 : 50, and 75 : 25). The highest *E*_r_ (91%) with a *C*_qe_ of 2.28 mg g^−1^ was obtained at a ratio of 75% NWC and 25% SWC, due to the combined effect of the high porosity of NWC and the structural stability of SWC.^46^ The other ratios (25 : 75 and 50 : 50) showed moderate performance, pointing out the need to optimize the natural-to-industrial carbon ratio in CCM.

In conclusion, NWC, SWC, and CCM are excellent adsorbents, and optimizing Ad_c_ and the adsorbent ratio can improve *E*_r_ while maintaining cost-effectiveness. The complete performance data for all adsorbents are summarized in Table 3.

#### 3.2.3. Effect of *t*_c_

**Fig. 12:**
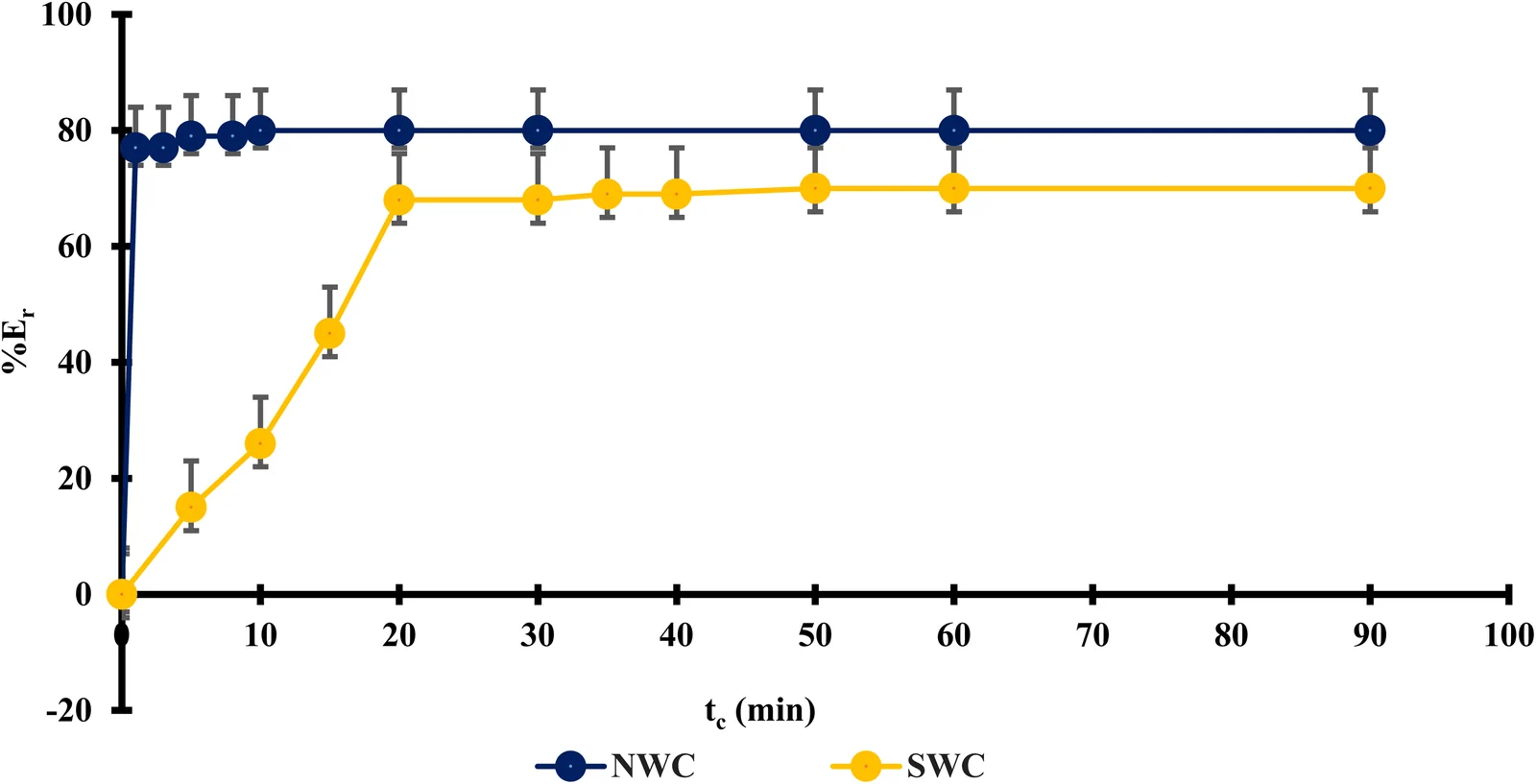
Illustrating the relationship between *t*_c_ and % *E*_r_. Error bars represent SD (*n* = 3).

**Fig. 13:**
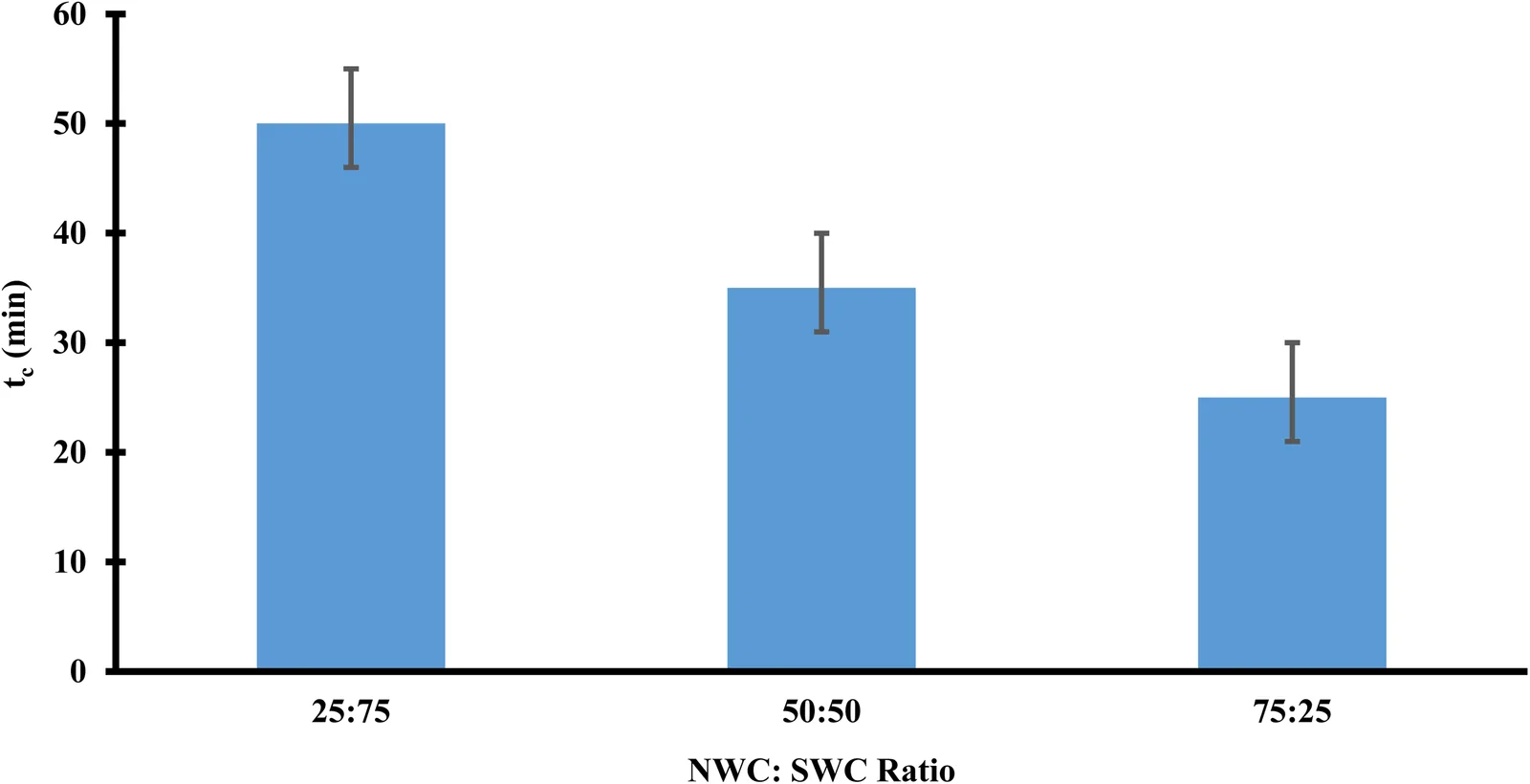
Impact on the *t*_c_ needed for MB_d_ adsorption of the composite ratio between NWC and SWC. Error bars represent SD (*n* = 3).

Using NWC, SWC, and CCM, Fig. 12 and 13 show the effect of *t*_c_ on the *C*_qe_ of MB_d_.

For NWC, the *C*_qe_ increased rapidly in the initial stage because of the high number of active sites, and equilibrium was reached within 10 min, achieving 89% *E*_r_ with a *C*_qe_ of 4.45 mg g^−1^. To confirm this fast adsorption process, the 10 minute time period was further divided into smaller time intervals (1, 3, 5, and 8 minutes), and it was confirmed that the *C*_qmax_ value was reached within this time. The high surface area, larger pores, and well-developed pore structure of NWC are in favor of the fast diffusion of dye molecules to the active sites.^47^

Conversely, SWC had a slower adsorption rate and reached equilibrium at 50 min with 75% *E*_r_ and *C*_qe_ of 3.50 mg g^−1^. This is attributed to its dense structure and small pore size, which restricts the diffusion of dye molecules to the adsorption sites.^48^

For CCM (Fig. 13) prepared with Ad_c_ of 0.2 g and different NWC : SWC ratios (25 : 75, 50 : 50, and 75 : 25), the equilibrium time was different for each composition. Equilibrium was reached at 50 min for the 25 : 75 ratio (75% *E*_r_, *C*_qe_ = 3.50 mg g^−1^), 35 min for the 50 : 50 ratio, and 25 min for the 75 : 25 ratio (91% *E*_r_, *C*_qe_ = 2.28 mg g^−1^). This clearly indicates the synergistic effect of the structural stability of SWC and the fast adsorption kinetics of NWC, implying that a higher content of NWC could speed up the adsorption process. The complete performance data are summarized in Table 3.

#### 3.2.4. Effect of *I*_c_

**Fig. 14:**
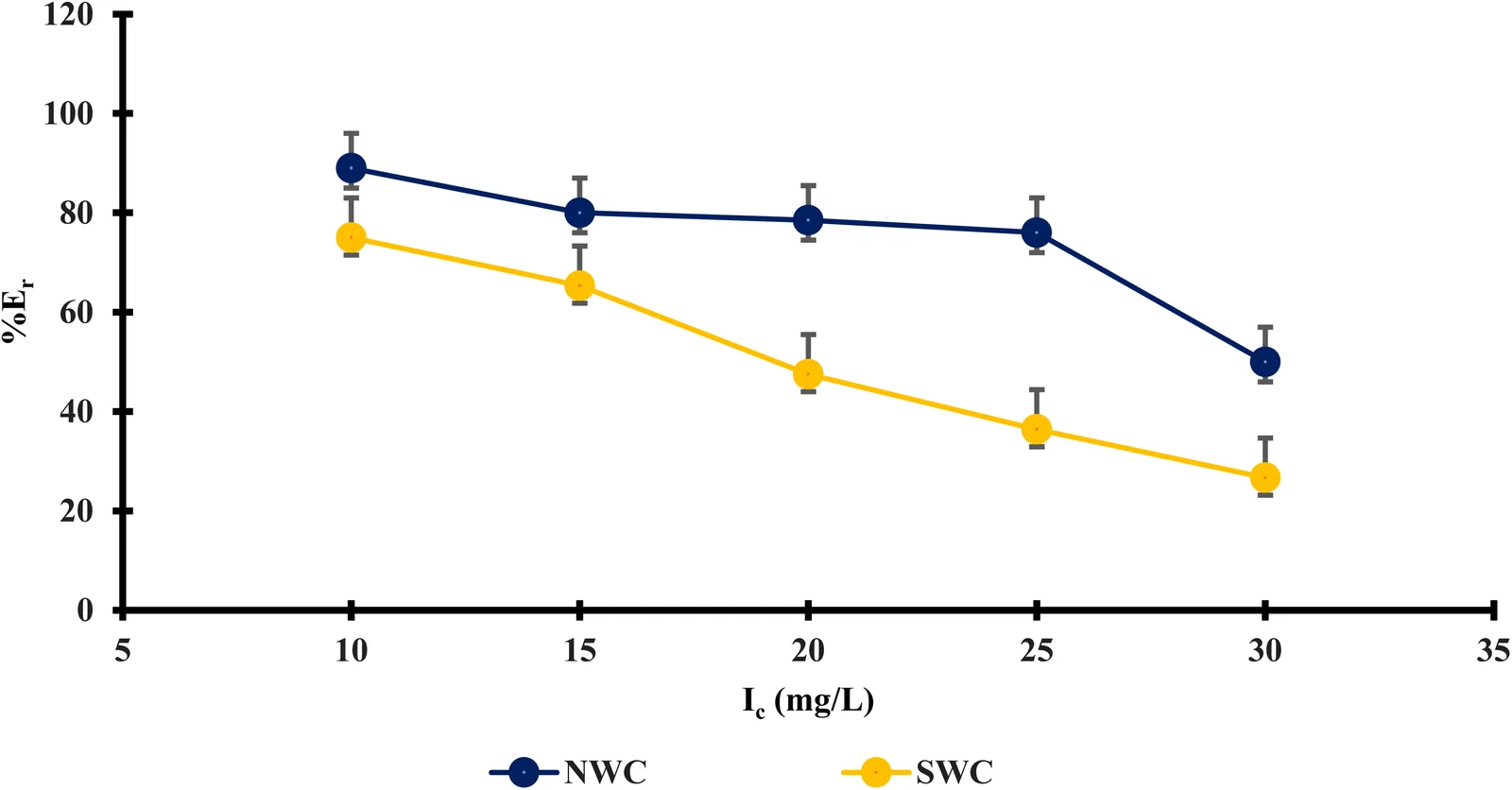
Illustrating the relationship between *I*_c_ and % *E*_r_. Error bars represent SD (*n* = 3).

The effect of the *I*_c_ on the MB_d_ adsorption performance using NWC and SWC is shown in Fig. 14. This increase in driving force for mass transfer between the dye solution and the adsorbent surface resulted in a higher *C*_qe_ with an increase in *I*_c_ from 10 to 30 mg L^−1^. For NWC, *C*_qe_ increased from 4.45 mg g^−1^ at *I*_c_ = 10 mg L^−1^ to higher values at higher concentrations. For SWC, *C*_qe_ also increased from 3.50 mg g^−1^ at *I*_c_ = 10 mg L^−1^. However, the percentage of dye *E*_r_ decreased at higher concentrations because the surface of the adsorbent was saturated with active sites, thus reducing the effectiveness.^49^

For both NWC and SWC, the optimum *I*_c_ for maximum *E*_r_ was 10 mg L^−1^, achieving 89% *E*_r_ with *C*_qe_ = 4.45 mg g^−1^ for NWC and 75% *E*_r_ with *C*_qe_ = 3.50 mg g^−1^ for SWC (see Table 3 for complete data). The dye was present at this concentration in sufficient quantities to occupy all the available active sites, without any saturation or competition between dye molecules for active sites. Although the absolute amount of dye adsorbed increased at higher concentrations, an optimum balance between uptake and available sites was achieved at 10 mg L^−1^.^8^ The results indicated that the lowest *I*_c_ gave the highest *E*_r_ for both carbon types. This indicates the importance of optimizing this parameter.

#### 3.2.5. Effect of *T*_c_

The effect of *T*_c_ on MB_d_ adsorption was investigated for both NWC and SWC at 30, 40, 50, and 60 °C. The results showed that *C*_qe_ decreased slightly for both adsorbents as *T*_c_ increased.^46^ The maximum *E*_r_ was achieved at 30 °C, with 89% *E*_r_ for NWC (*C*_qe_ = 4.45 mg g^−1^) and 75% *E*_r_ for SWC (*C*_qe_ = 3.50 mg g^−1^) (see Table 3 for complete data), indicating that the adsorption process is exothermic.

The decrease in adsorption at higher *T*_c_ can be explained by the increase in the kinetic energy of dye molecules, which may cause the adsorbed molecules to desorb from the surface of the adsorbent. Moreover, the reduction in *E*_r_ is due to the reduction in the electrostatic and van der Waals interactions between the dye molecules and the adsorbent surface at high *T*_c_.^46,50^

**Fig. 15:**
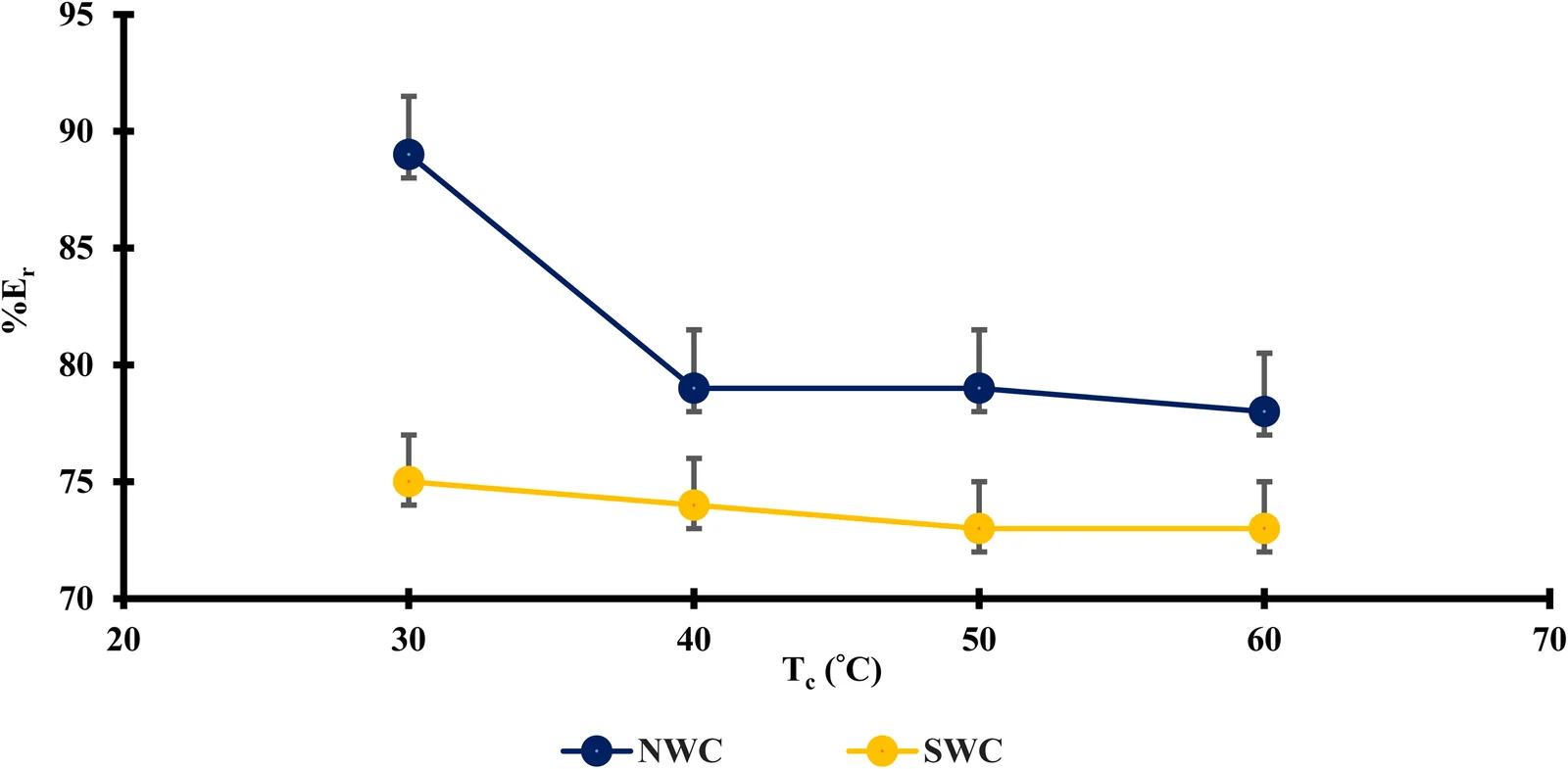
Illustrating the relationship between *T*_c_ and % *E*_r_. Error bars represent SD (*n* = 3).

For both biomass-derived NWC and industrial textile-derived SWC, the best condition to achieve the highest *E*_r_ was a moderate *T*_c_ of 30 °C. These results suggest that both ACs exhibit more effective MB_d_ adsorption at lower *T*_c_, which makes them suitable for practical wastewater treatment under ambient conditions without additional heating.^50^ These results are shown in Fig. 15.

#### 3.2.6. Summary of optimal adsorption conditions

Table 3 summarizes the optimal adsorption conditions and corresponding performance for NWC, SWC, and CCM. Generally, NWC showed the fastest adsorption kinetics (10 min) with high *E*_r_ (89%, *C*_qe_ = 4.45 mg g^−1^), while SWC showed slower kinetics (50 min) and lower *E*_r_ (75%, *C*_qe_ = 3.50 mg g^−1^). The CCM, with its optimized composition (75 : 25 NWC : SWC), achieved the highest *E*_r_ (91%, *C*_qe_ = 2.28 mg g^−1^) with a balanced *t*_c_ of 25 min. All adsorbents showed optimal performance at room temperature (30 °C) and an *I*_c_ of 10 mg L^−1^. The C_qmax_ derived from the Langmuir model is also included in Table 3 for comparison.

### 3.3. pH_zcp_ determination of NWC, SWC, and CCM adsorbents

**Fig. 16:**
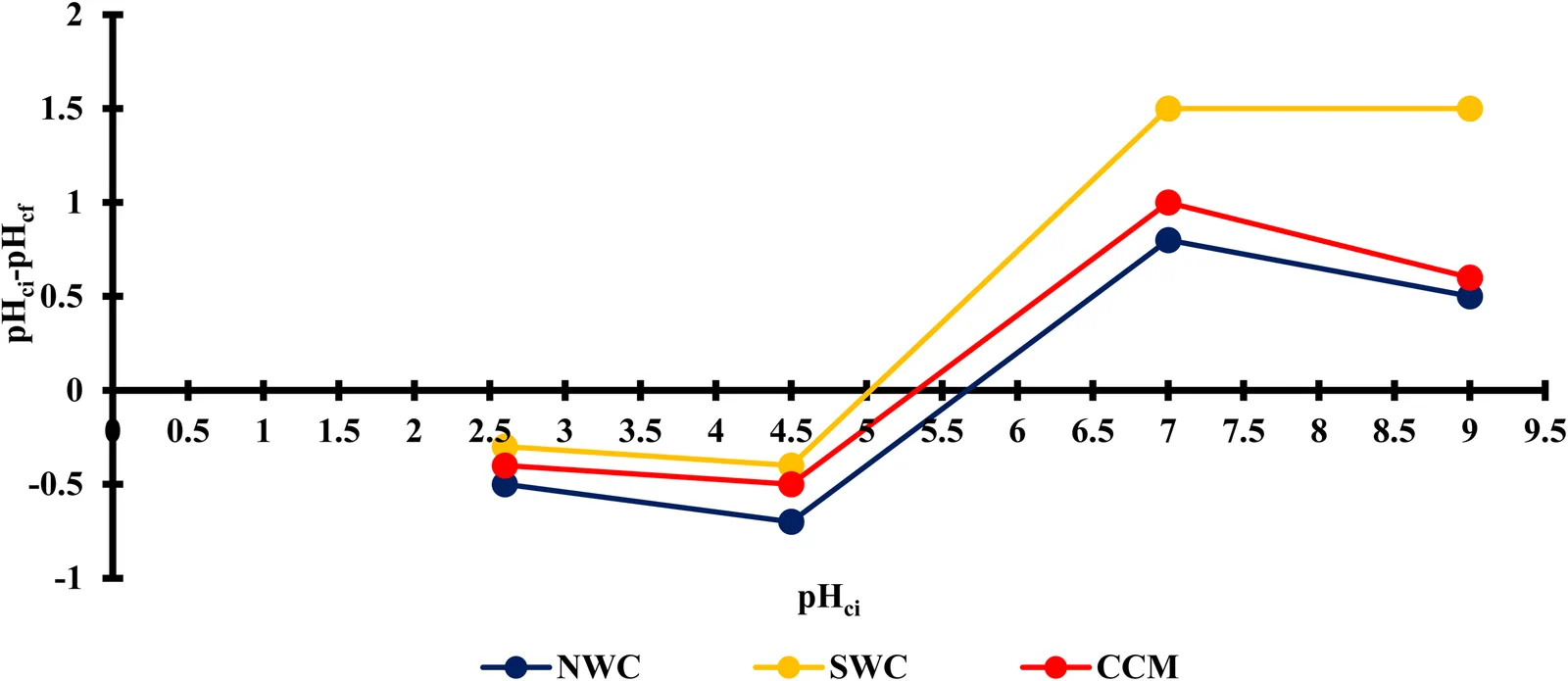
Shows the pH_c_ drift curves for the determination of pH_zcp_ of the three prepared adsorbents: NWC, SWC, and CCM.

The pH_c_ drift curves of Fig. 16 clearly show differences in the pH_zcp_ among the three carbon adsorbents. The negative ΔpH_c_ at low pH_ci_ = 2–4 indicated the positively charged surface of adsorbents, which could easily gain.^4^ With increasing pH_c_, ΔpH_c_ gradually approached zero, and the pH_zcp_ for each sample was determined at the pH_c_ at which the curve crossed the zero line. The highest pH_zcp_ (approximately 5.2) was found in the SWC sample. This indicated that the SWC sample was more alkaline and presented fewer acidic oxygen functional groups. The CCM sample had a medium pH_zcp_ at a pH_c_ of approximately 5.0, which suggests the balance between acidic and basic functionalities. The NWC sample has the lowest pH_c_ value of approximately 4.8, which means that it has the highest amount of acidic groups, such as carboxyl (–COOH) and hydroxyl (–OH) groups, which are more prone to losing protons. Due to the high level of deprotonation of the acidic groups, the ΔpH_c_ values of the adsorbents became progressively positive at pH_c_ values above 6–7, indicating the presence of a negative surface charge.^4^ SWC had the highest ΔpH_c_ in the alkaline range, which was consistent with its pH_zcp_ values and lower level of acidity. It was observed that surface chemistry depends to a large extent on the nature of the precursors and the method of activation employed for the preparation of the materials. The pH_zcp_ values obtained for all the adsorbents indicated that all the adsorbents would be negatively charged at the pH_c_ values for wastewater, which would be between 6 and 9, favoring the adsorption of cationic dyes while having a lesser affinity for anionic dyes.

### 3.4. Kinetic models analysis

**Fig. 17:**
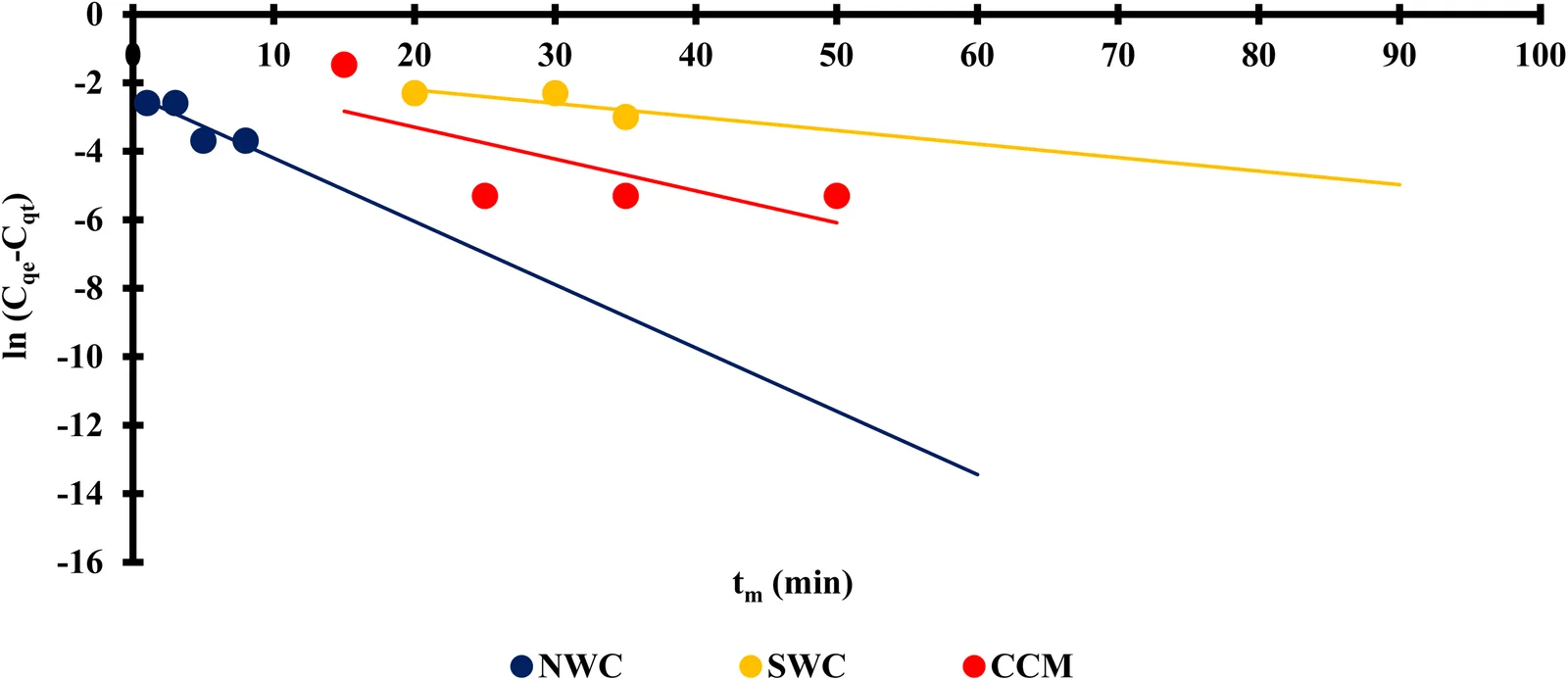
PFO_m_ kinetics for MB_d_ adsorption onto NWC, SWC, and CCM at different *t*_m_ intervals.

**Fig. 18:**
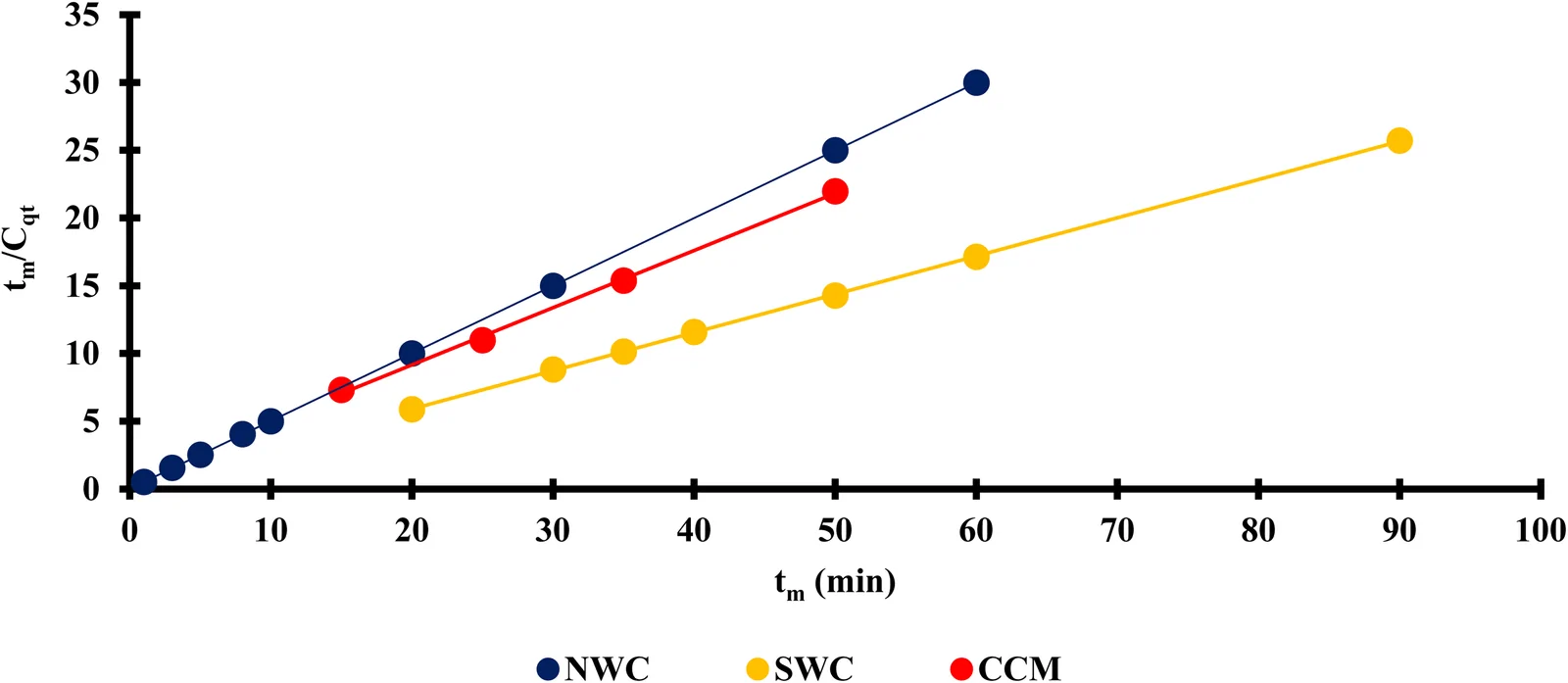
PSO_m_ kinetics for MB_d_ adsorption onto NWC, SWC, and CCM at different *t*_m_ intervals.

The adsorption kinetics of MB_d_ onto NWC, SWC, and CCM were studied using PFO_m_ and PSO_m_. Fig. 17 shows the PFO_m_ plots for the three adsorbents, while Fig. 18 shows the PSO_m_ plots.

The PFO_m_ showed poor applicability, as indicated by low *R*^2^ between 0.53 and 0.76 and large discrepancies between the calculated (*C*_qe_, cal) and experimental (*C*_qe_, exp) values.^51^ This finding indicates that the PFO_m_ does not adequately describe the adsorption process for any of the tested adsorbents.

In contrast, the PSO_m_ fitted the experimental data very well for all adsorbents, with *R*^2^ values very close to 1 (0.998–0.9999) and calculated *C*_qe_,cal values in excellent agreement with experimental *C*_qe_,exp values, confirming that the PSO_m_ best describes MB_d_ adsorption.^8^ The kinetic constants were different among the adsorbents. The highest *k*_2_ (7.75 g mg^−1^ min^−1^) was for NWC because of its high porosity and large surface area, which enable it to adsorb a large amount of dye in a short time. The lowest *k*_2_ (0.299 g mg^−1^ min^−1^) was for SWC, which was in line with the denser pore structure and slower adsorption rate. The intermediate *k*_2_ of CCM (0.244 g mg^−1^ min^−1^) indicated the intermediate adsorption rate because of the combined characteristics of NBW and SFW.

The good fit with the PSO_m_ indicates that the rate-limiting step may be chemisorption. To support this conclusion, the Δ*H*_s_ were also investigated. NWC (−21.15 kJ mol^−1^) and CCM (−39.72 kJ mol^−1^) have Δ*H*_s_ values within the range of normal chemisorption (−20 to −80 kJ mol^−1^) as shown in Table 7. The value of Δ*H*_s_ of SWC (−10.89 kJ mol^−1^) shows that the process is a combination of physisorption and chemisorption. Additionally, the FTIR studies before and after adsorption confirmed the involvement of oxygen-containing functional groups (–OH, –COOH, C=O) in the adsorption process, which further supports the chemical interactions.

The kinetic parameters also give insight into the nature of adsorption. The high value of *k*_2_ for NWC (7.75 g mg^−1^ min^−1^) can be attributed to the highly developed macroporous and mesoporous nature of the material (as revealed by BET and SEM analyses), which provides easy access for dye molecules to the material's active sites. The lower value of *k*_2_ for SWC (0.299 g mg^−1^ min^−1^) indicates a microporous and dense structure, which impedes molecular diffusion; the moderate value of *k*_2_ for CCM (0.244 g mg^−1^ min^−1^) indicates an intermediate pore structure, which is an ideal compromise of the two features, with fast kinetics as NWC but with the stability of SWC.

Kinetic parameters for all adsorbents are summarized in Table 4. The kinetics obtained graphically also support the reliability of the PSO_m_ with respect to the PFO_m_.^52^

**Table 4:** Comparison of kinetic parameters for MB_d_ adsorption onto the three adsorbents

Adsorbent	NWC	SWC	CCM
*C*_qe,exp_ (mg g^−1^)	2.00	3.50	2.28
PFO_m_*k*_1_ (min^−1^)	0.185	0.040	0.093
PFO_m_*C*_qe_, calc. (mg g^−1^)	0.095	0.244	0.238
PFO_m_*R*^2^	0.76	0.71	0.53
PSO_m_*k*_2_ (g mg^−1^ min^−1^)	7.75	0.299	0.244
PSO_m_*C*_qe_, calc. (mg g^−1^)	2.00	3.54	2.37
PSO_m_*R*^2^	0.9999	0.998	0.9997

### 3.5. Adsorption isotherm analysis

**Fig. 19:**
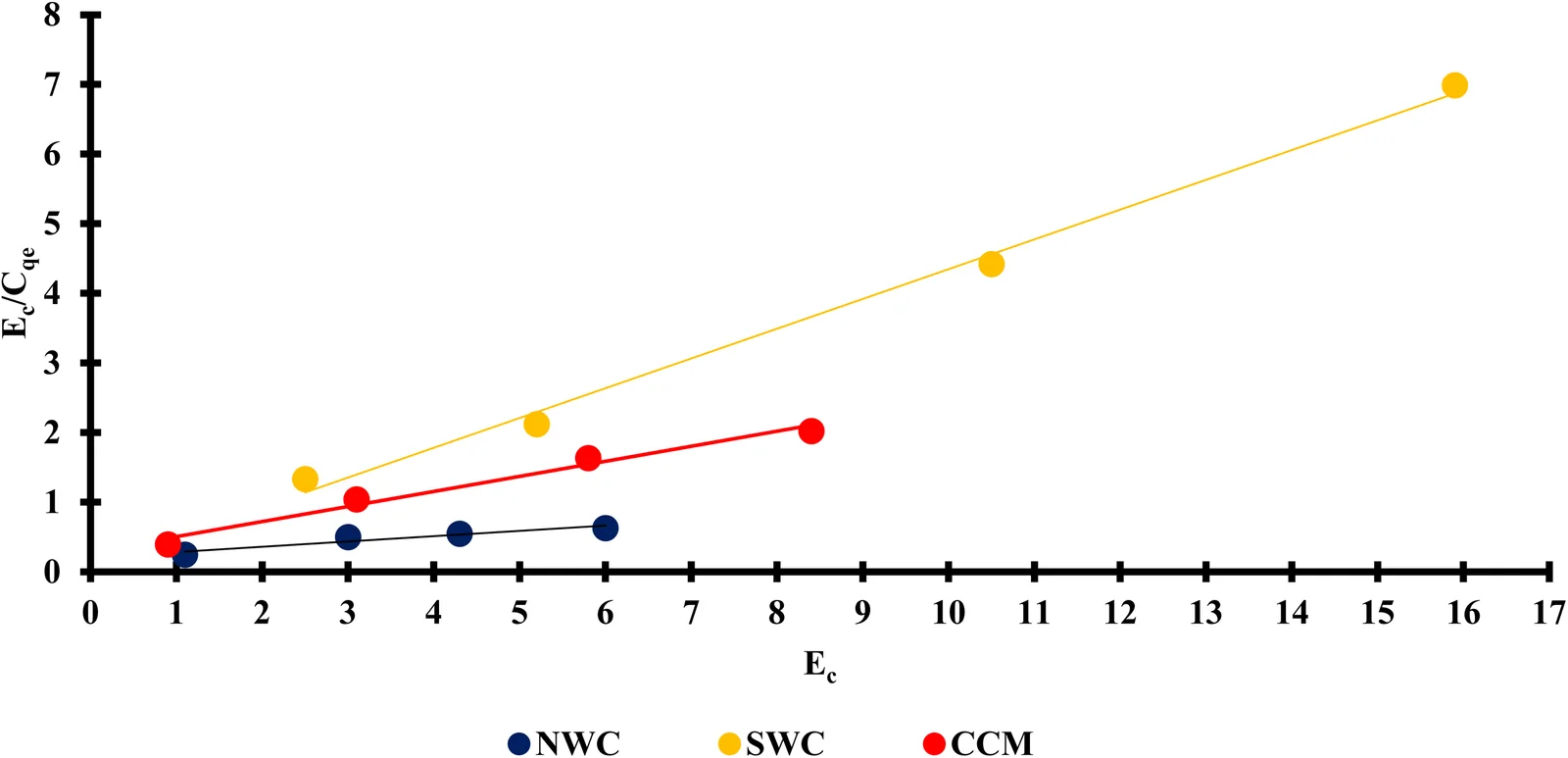
Langmuir isotherm plots for NWC, SWC, and CCM showing monolayer adsorption behavior.

**Fig. 20:**
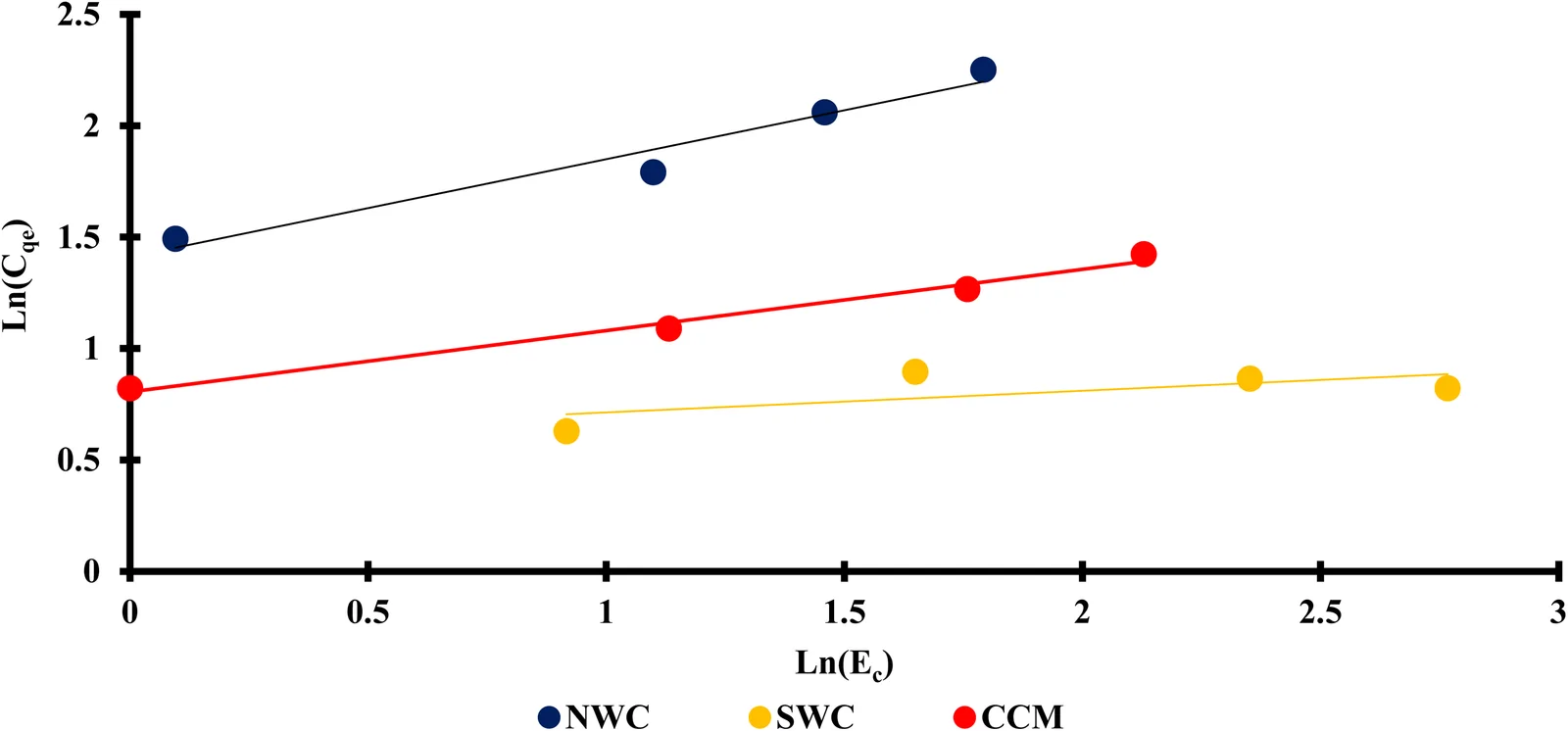
Freundlich isotherm plots for NWC, SWC, and CCM confirming heterogeneous surface adsorption.

The goodness-of-fit of each model was evaluated using the *R*^2^, with values closer to 1 indicating a better fit. The kinetic and isotherm parameters with *R*^2^ values are listed in Tables 4 and 5, respectively. The adsorption characteristics of the three adsorbents, including NWC, SWC, and CCM, were assessed *via* the Langmuir and the Freundlich isotherm models. As illustrated in Fig. 19, the Langmuir plots showed good linear correlation, especially for SWC and CCM, indicating that the adsorption process predominantly follows a monolayer adsorption mechanism. The *C*_qmax_ values derived from the Langmuir model were 13.11, 2.34, 25.45 mg g^−1^ for NWC, SWC, and CCM, respectively, demonstrating the better adsorption efficacy of CCM as compared with the other adsorbents. Meanwhile, the Freundlich plots in Fig. 20 indicated that NWC and CCM fit well with the Freundlich model (*R*^2^ = 0.9544 and 0.9875, respectively), suggesting heterogeneous and multilayer adsorption, whereas SWC showed weak fitting (*R*^2^ = 0.4321). The summarized isotherm parameters in Table 5 indicate that CCM exhibited the strongest *C*_qmax_, followed by NWC, whereas SWC showed the weakest. It is observed that CCM fits well with Langmuir (*R*^2^ = 0.9806) and Freundlich (*R*^2^ = 0.9875) models. The dual behavior is not inconsistent but a manifestation of the mixed porous structure of CCM. The micropores derived from the SWC favor the monolayer adsorption on relatively homogeneous surfaces (Langmuir behavior), while the mesopores derived from the NWC favor the multilayer adsorption on heterogeneous surfaces (Freundlich behavior). CCM derived from two different precursors exhibit dual-isotherm behavior. The properties of the tested carbon strongly influence the variations in *C*_qmax_. CCM has a well-developed pore structure and a large specific surface area, which means it has more places for adsorption, as noted in a recent study about NWC, which also has a high surface area and complex pore structures.^31^ The average performance of NWC is attributed to its porosity and the presence of oxygen- and nitrogen-containing groups from biomass, which enhance its ability to attract and interact with adsorbates.^53^ The adsorption performance of CCM was compared with the recently reported adsorbents in Table 6. It compares important parameters such as activation method, surface area, pore volume, regeneration cycles, and *C*_qmax_. CCM shows a competitive surface area (510 m^2^ g^−1^) and pore volume (0.487 cm^3^ g^−1^) compared with other adsorbents, with a *C*_qmax_ of 25.45 mg g^−1^, better than single-precursor adsorbents, such as date pit-derived AC (∼18 mg g^−1^).^18^ it is comparable to olive waste-derived AC (34.3 mg g^−1^)^2^ and to clay-carbon composites (∼18.5 mg g^−1^).^10^ And chitosan/activated carbon composite (∼22.5 mg g^−1^).^45^ Although some biomass-derived carbons, such as papaya stem-derived AC (990 mg g^−1^),^4^ exhibit higher capacities, CCM provides the unique advantage of utilizing mixed waste sources (NBW and SFW) with a tailored synthesis approach, contributing to waste valorization and circular economy principles. Moreover, good reusability of CCM over four regeneration cycles was demonstrated, further supporting its practical applicability. The isotherms also give more information about the mechanism of adsorption. The values of *n*_*i*_ for NWC (1/*n*_*i*_ = 0.44) and CCM (1/*n*_*i*_ = 0.27) are within the range of 0–1, which indicates the favorable adsorption process on the heterogeneous surfaces. The higher value of *K*_F_ for CCM (6.39) than NWC (4.09) and SWC (1.85) shows a greater extent of adsorption at different concentrations. The *K*_L_ of SWC (6.08 L mg^−1^) confirms a high affinity between the adsorbent and MB_d_ despite its low surface area. The double nature of CCM, where it fits Freundlich and Langmuir, is a result of its mixed pore nature, whereby the micropores formed by SWC facilitate monolayer adsorption and the mesopores formed by NWC facilitate multilayer adsorption.

**Table 5:** Langmuir and Freundlich parameters for the three adsorbents

Adsorbent	Langmuir model			Freundlich model		
	*C*_qmax_	*K*_L_	*R* ^2^	*K*_F_	1/*n*_*i*_	*R* ^2^
NWC	13.11	0.369	0.9113	4.09	0.44	0.9544
SWC	2.34	0.090	0.9948	1.85	0.1	0.4321
CCM	25.45	0.746	0.9806	6.39	0.27	0.9875

**Table 6:** Comparison of activation methods, surface properties, reusability, and *C*_qmax_ of CCM with recently reported waste-derived ACs for MB_d_ removal

Adsorbent	Activation method	Surface area (m^2^ g^−1^)	Pore volume (cm^3^ g^−1^)	Regeneration cycles	Key distinguishing point	*C*_qmax_ (mg g^−1^)	Reference
Papaya-based carbon	H_3_PO_4_	Stems: 1053.52	Stems: 1.1580	5 cycles for SAC (no significant efficiency loss)	The first study to utilize different parts of the same plant (stems and leaves) as different feedstocks with significantly different porous properties and very high adsorption capacities, especially for stems	∼642–990	4
		Leaves: 441.67	Leaves: 0.4858				
Olive waste-derived AC	KOH	934.93	0.456	Not reported	Best conditions: 700 °C, 1 : 1 impregnation ratio (KOH : char), 60 min activation time → MB_d_ removal up to 99% at pH 10, 25 °C, 100 mg L^−1^*I*_c_	34.3	2
Chitosan/activated carbon composite	—	Not specified	Not specified	Not reported	Focus on chemical resistance (wide pH range) and mechanical strength (easy separation, fixed-bed applicability)	∼22.5	45
Clay-carbon composite	—	Not specified	Not specified	Not reported	A comprehensive review covering research evolution from 2005 to 2025 highlights the shift from traditional AC to advanced composites with high adsorption capacities, selective recognition, and catalytic degradation capabilities	∼18.5	10
Date pits-derived AC	ZnCl_2_	Not specified	Not specified	Not reported	High adsorption capacities for BG (247.75 mg g^−1^) through multiple mechanisms: pore-filling, electrostatic attraction, H-bonding, and π–π stacking	∼18	18
CCM	NaOH/HCl	510	0.487	4 cycles tested	Dual-waste composite; precursor-specific synthesis; optimized composition; tested on real textile wastewater	25.45	This work

### 3.6. Thermodynamic studies

**Fig. 21:**
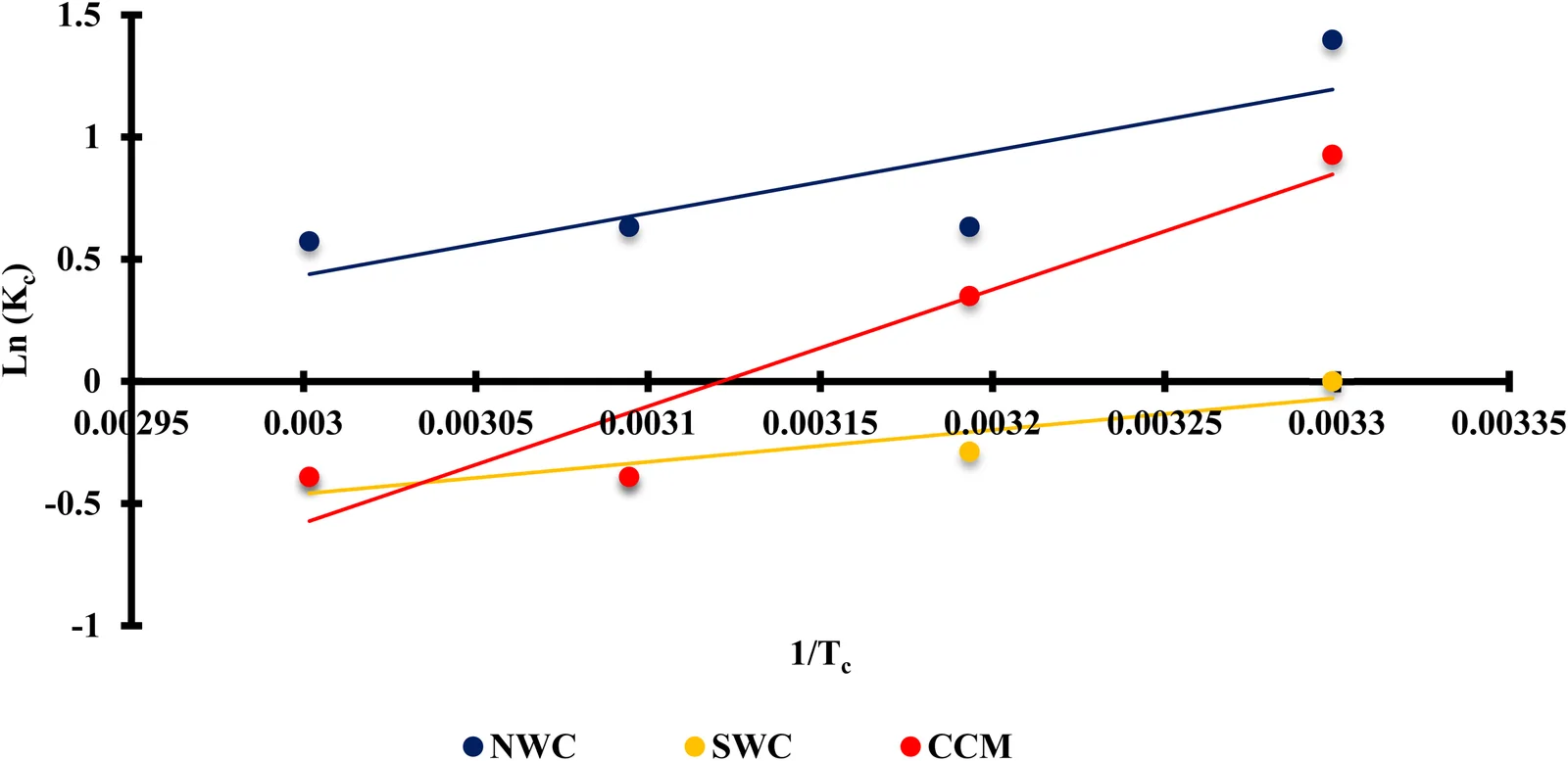
Thermodynamic van't Hoff plots for MB_d_ adsorption onto NWC, SWC, and CCM at different *T*_c_.

The thermodynamic factors (Δ*G*_s_, Δ*H*_s_, and Δ*S*_s_) were examined to learn how MB_d_ sticks to the created adsorbents. For NWC, the values of Δ*G*_s_ were negative, from −3522.48 J mol^−1^ to −1585.77 J mol^−1^ which confirmed the spontaneity of the adsorption process. The change in Δ*H*_s_ is −21.15 kJ mol^−1^ which implies an exothermic reaction, while the negative value of Δ*S*_s_, −59.83 J mol^−1^ K^−1^ implies the decrease in disorder. However, the Δ*G*_s_ values for CCM were negative at lower *T*_c_ (−2337.26 to −906.82 J mol^−1^) confirming spontaneous adsorption but shifted to positive values (+1052.29 and +1084.85 J mol^−1^) at higher *T*_c_, indicating that the spontaneity decreased with the rise of *T*_c_. The Δ*H*_s_ = −39.72 kJ mol^−1^ was more negative than the individual carbons, suggesting that the interactions were more exothermic. The Δ*H*_s_ values obtained provide further insight into the adsorption mechanism. The values of NWC (−21.15 kJ mol^−1^) and CCM (−39.72 kJ mol^−1^) are consistent with chemisorption, whereas the lower value of SWC (−10.89 kJ mol^−1^) indicates mainly physisorption. Also, the Δ*S*_s_ = −123.97 J mol^−1^ K^−1^ indicated a significant reduction in the disorder at the interface.^54^ In summary, spontaneous adsorption is most favorable for NWC and least favorable for SWC due to weak non-spontaneous adsorption. Concurrently, CCM exhibits the characteristics of both, offering better adsorption performance at lower *T*_c_ but less spontaneity at higher *T*_c_. A summary of the obtained thermodynamic parameters for all adsorbents is given in Table 7, and the change of Δ*G*_s_ with Tc for NWC, SWC, and CCM is shown in Fig. 21.

**Table 7:** Thermodynamic parameters (Δ*G*_s_, Δ*H*_s_, and Δ*S*_s_) of MB_d_ adsorption onto NWC, SWC, and CCM

Adsorbent	Δ*G*_s_ range (J mol^−1^)	Δ*H*_s_ (kJ mol^−1^)	Δ*S*_s_ (J mol^−1^ K^−1^)
NWC	−3522.48 to −1585.77	−21.15	−59.83
SWC	0 to +1084.85	−10.89	−36.48
CCM	−2337.26 to +1084.85	−39.72	−123.97

## 4. Application to real textile wastewater

The synthesized adsorbents were evaluated for their performance by batch adsorption tests in real textile effluent obtained from Startex Company, as described in Table 1.

### 4.1. Comparison of performance

Under optimized conditions, the highest *E*_r_ was observed in CCM (75%), followed by NWC (65%), CAC (62%), and SWC (55%). The overall performance comparison is summarized in Table 8.

**Table 8:** Comparison of % *E*_r_ of MB_d_ by NWC, SWC, CCM, and CAC in synthetic *versus* real textile wastewater. Values are presented as mean ± SD (*n* = 3)

Adsorbent	Synthetic MB_d_ removal (%)	Real wastewater *E*_r_ (%)	Drop (%)
NWC	89 ± 2.1	65 ± 3.2	24
SWC	75 ± 1.8	55 ± 2.5	20
CCM	91 ± 1.5	75 ± 2.8	16
CAC	85 ± 2.0	62 ± 3.0	23

### 4.2. Effect of wastewater composition

The reduction of *E*_r_ from synthetic MB_d_ solution (91% for CCM) to real wastewater (75% for CCM) can be attributed to the complex composition of real textile effluent. The real wastewater contains high concentrations of COD (3300 mg L^−1^), BOD (2000 mg L^−1^), TSS (520 mg L^−1^), and *I*_c_ of dye (80 mg L^−1^), as shown in Table 1. These competing organic pollutants can even occupy the active adsorbing sites, whereas the TSS may physically block the porous structure of the adsorbent. Moreover, the presence of salt and surfactants that are usually found in textile wastewater can reduce the electrostatic attraction between the positively charged MB_d_^+^ ions and the negatively charged adsorbent surface, leading to a reduction of the overall *E*_r_.^9,10^

### 4.3. Practical applicability and regeneration

Despite the decrease in efficiency from synthetic to real wastewater, CCM still retained 75% *E*_r_, which remained higher than all other adsorbents tested (NWC: 65%, SWC: 55%, CAC: 62%). This indicates that CCM is suitable for treating real wastewater with COD levels up to 3300 mg L^−1^ and TSS up to 520 mg L^−1^. For wastewaters with higher pollutant loads, pretreatment steps such as filtration or dilution are recommended.

**Fig. 22:**
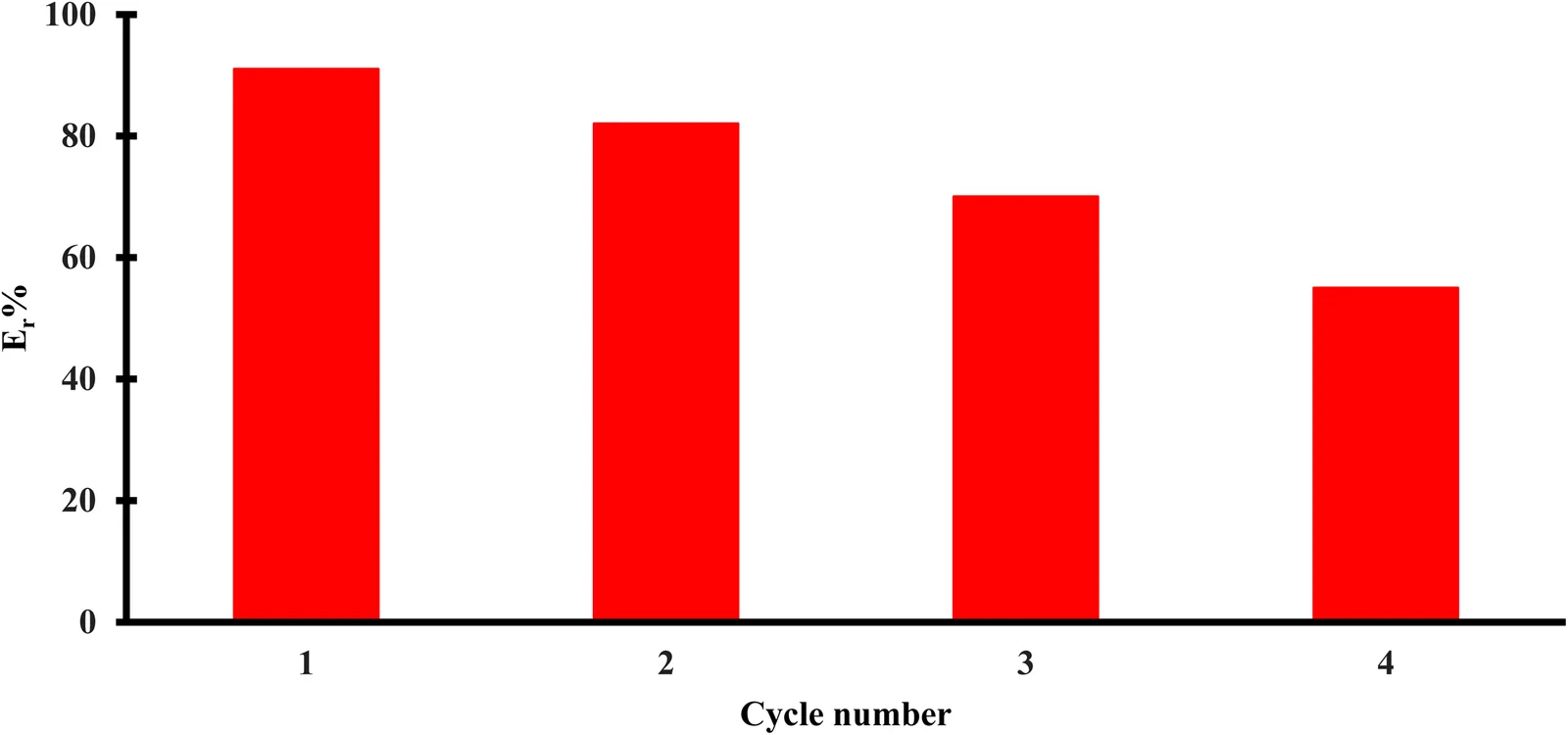
Reusability of CCM for four consecutive adsorption–desorption cycles using 0.1 M HCl as the regenerating agent. Error bars represent SD (*n* = 3).

The reusability of CCM was evaluated by performing four successive adsorption–desorption cycles, as shown in Fig. 22. Between cycles, the spent adsorbent was regenerated by treating it with a 0.1 M HCl solution, washing it with DI water to neutral pH, and drying it at 80 °C for 6 hours. The *E*_r_ decreased from 91% in the first cycle to 82%, 70%, and 55% in the second, third, and fourth cycles, respectively, as shown in the graph. This is due to the incomplete regeneration of active sites and the complete desorption of MB_d_ molecules from the adsorbent. However, CCM retained about 70% of its original capacity even after three adsorption cycles.

## 5. Adsorption mechanism

The adsorption mechanism of MB_d_ onto the prepared ACs is a combination of several simultaneous interactions, which were elucidated through kinetic modeling, thermodynamic analysis, FTIR spectroscopy, pH_zcp_ measurements, and BET surface area analysis (summarized in Tables 2, 3, 4, and 7 and illustrated in Fig. 17–22). This multi-mechanistic process is in line with recent findings, where electrostatic attractions, π–π interactions, and hydrogen bonding have been identified as the main driving forces for MB_d_ adsorption onto carbonaceous materials.^55^ Moreover, the synergistic effect of these interactions has been confirmed for CCM.^56^

### 5.1. Electrostatic attraction

The pH_zcp_ of NWC, SWC, and CCM are 4.8, 5.2, and 5.0, respectively, which indicates the surface of the adsorbents is negatively charged at the working pH_c_ (6–7). This negative charge is advantageous for strong electrostatic attraction with the positively charged MB_d_^+^ cations, thus facilitating the *E*_r_ of the dye.^57^ The higher surface charge density of CCM is responsible for its better *E*_r_ (91%) than SWC (75%). This is consistent with the pH_c_-dependent adsorption behavior shown in Fig. 9.^58^

### 5.2. π–π interactions

The aromatic rings of MB_d_ molecules are involved in π–π stacking with the delocalized π–electron system of the graphitic carbon layers.^59^ These interactions are of particular importance for CCM, which possesses a more ordered carbon structure due to the contribution of the SWC component, as revealed by the XRD patterns in Fig. 7B. These interactions are also supported by the presence of aromatic C=C bands in the FTIR spectra (Fig. 4B).^60^

### 5.3. Hydrogen bonding

FTIR analysis showed that oxygen functional groups (O–H, C=O, and C–O) were present on the surfaces of NWC and CCM (Fig. 4a and b). The groups form hydrogen bonds with the nitrogen atoms and amine groups of the MB_d_ molecules, which further enhances the adsorption process.^11^ The higher efficiency of CCM compared to SWC is attributed to the retention of these functional groups in CCM. This finding is in agreement with FTIR results presented in Section 3.1.2.^61^

### 5.4. Pore filling

The BET results (Table 2) showed that CCM had the largest surface area (510 m^2^ g^−1^) and a well-developed pore structure with an adequate combination of micro- and mesopores.^62^ This dual porosity enables efficient pore filling where MB_d_ molecules are trapped in pores. The mesopores enable fast diffusion, and the micropores provide additional binding sites that result in the overall high adsorption performance.^63^ The pore size distribution is shown in Fig. 6.

### 5.5. Kinetic and thermodynamic support

The good agreement between the PSO_m_Table 4 and Fig. 18 indicates that the rate-limiting step is likely to be chemisorption, which is supported by the presence of oxygen-containing functional groups as identified by FTIR.^64^ Moreover, the thermodynamic parameters (Table 7 and Fig. 21) show that the adsorption process is spontaneous and exothermic.^65^ The Δ*H*_s_ values of NWC (−21.15 kJ mol^−1^) and CCM (−39.72 kJ mol^−1^) are in the range of typical chemisorption (−20 to −80 kJ mol^−1^), whereas SWC (−10.89 kJ mol^−1^) suggests a mixed physisorption-chemisorption mechanism.^66^

### 5.6. Combined effect

**Fig. 23:**
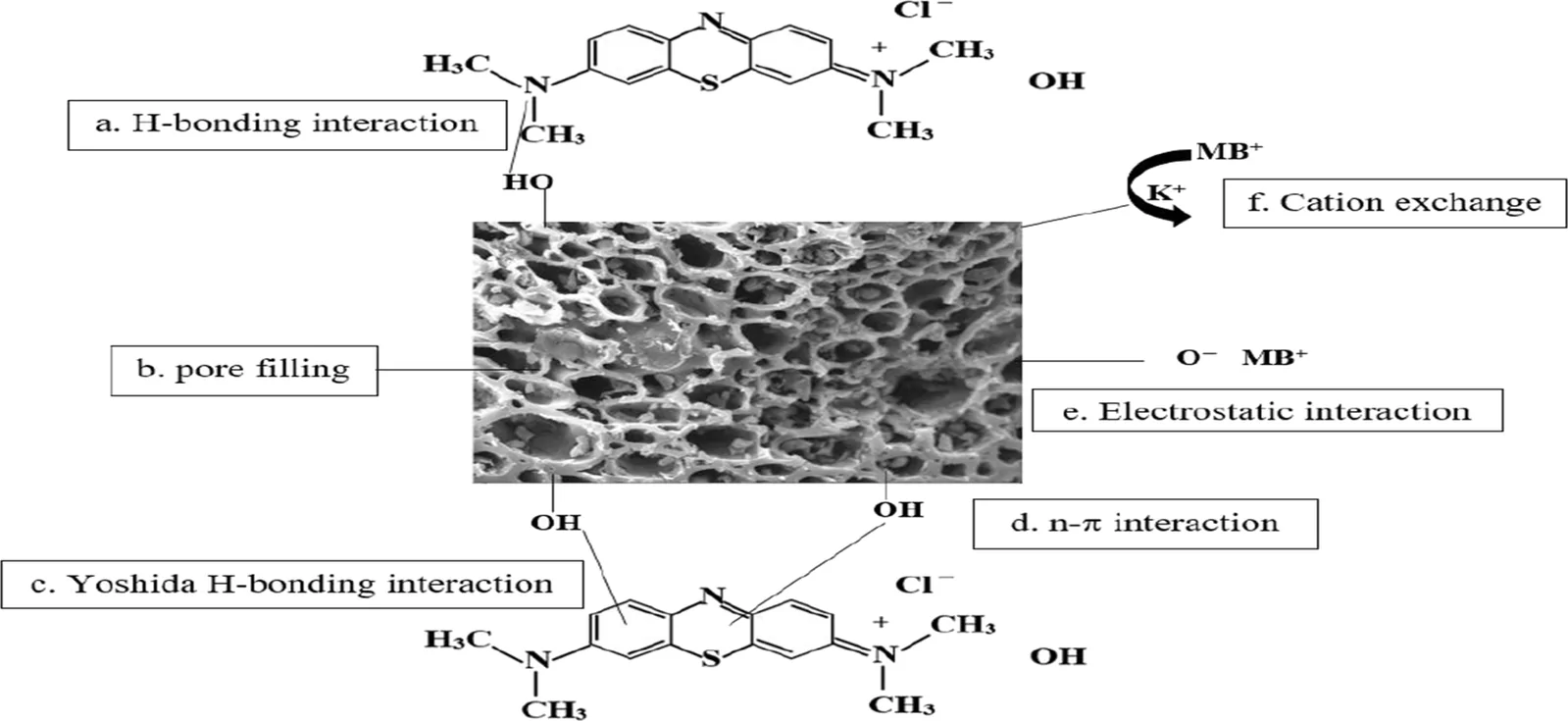
Proposed mechanisms of MB_d_ adsorption onto NWC, SWC, and CCM, including electrostatic attraction, π–π interactions, hydrogen bonding, and pore filling.

This is due to the synergistic effects of these mechanisms that resulted in a better performance of CCM (91% *E*_r_).^67^ The CCM uses the oxygen-containing functional groups of the NWC (improving electrostatic attraction and hydrogen bonding), the aromatic structure of SWC (enhancing π–π interactions), and the balanced pore structure (allowing pore filling and fast diffusion).^68^ Thus, the multi-mechanism adsorption process is responsible for the more effective adsorption by CCM than the individual adsorbents, as clearly shown in Table 7 and Fig. 23.

## 6. Conclusion

The present study demonstrates a novel and sustainable route for the preparation of a CCM from two different waste streams, namely NBW and SFW. Unlike most previous studies that focused on single-precursor carbons, this work combined both waste-derived carbons in one composite material, systematically optimized the NWC : SWC ratio, and verified the performance of the optimized CCM not only in synthetic MB_d_ solutions but also in real textile wastewater. In this study, ACs were successfully prepared from NBW and SFW to produce NWC, SWC, and CCM. The properties of these ACs were also characterized; hence, it was found that the CCM had the highest surface area of 510 m^2^ g^−1^. Batch adsorption tests revealed that NWC exhibited fast adsorption kinetics, achieving 89% *E*_r_ within 10 min, whereas SWC showed a slower rate, achieving 75% removal after a longer contact time. The CCM had a high degree of 91% *E*_r_ under optimal conditions due to the synergistic effect of both precursors. The isotherm and kinetic analyses revealed multilayer adsorption for NWC (Freundlich) and monolayer adsorption for SWC and CCM (Langmuir), and the PSO_m_ was fitted to all adsorbents, confirming chemisorption as the dominant process. The practical efficiency of CCM in dye removal was also demonstrated on real textile wastewater with 75% removal. This method is more efficient in dye removal than single carbons and CAC. All in all, CCM is a clean and efficient technique for dye removal from wastewater with a high surface area, fast adsorption, and high efficiency. The PSO_m_ kinetics combined with thermodynamic parameters indicates that the main mechanism in MB_d_^+^ removal is chemisorption, especially for NWC and CCM, but SWC shows a mixed mechanism of physisorption-chemisorption.

The superiority of CCM over NWC and SWC could be attributed to its mixed porous structure, which integrates the merits of NWC (high surface area and mesopores) and SWC (microporosity and structural stability). This mixed porous structure could provide more diverse adsorption sites and faster kinetics. Most of the previous studies were focused on single-precursor ACs tested on synthetic dye solutions. The present work demonstrates that a CCM derived from mixed waste (NBW + SFW) can achieve high *E*_r_ (91%) and also perform effectively on real textile wastewater, pointing towards its practical relevance and contribution towards waste valorization.

However, despite these promising results, some limitations need to be acknowledged. The study was limited to batch adsorption experiments. Further studies are needed to assess the performance of the adsorbent in continuous flow systems (column studies). The regeneration studies were also limited to four cycles, and it is recommended that future work examine stability and cost-effectiveness over longer time scales. The dual waste valorization approach of the CCM is presented as economically and sustainably viable by converting zero-cost domestic and industrial wastes into a high-performance material. Quantitatively, the CCM (75% *E*_r_) is found to be superior to CAC (62% *E*_r_) for treating real textile effluent, being a more economical solution to complex industrial matrices. Moreover, the CCM can maintain 70% of its capacity after three regeneration cycles under optimal operating conditions at ambient temperature (30 °C) to cut down the cost of replacement and energy consumption. The CCM is more efficient than some of the single-precursor adsorbents recently reported, with a *C*_qmax_ of 25.45 mg g^−1^, and thus a scientifically and economically sound alternative for sustainable water remediation.

## Author contributions

SM, AS, MM, and AES initiated the study. SM, AS, MM, and AES collected the materials and drafted the manuscript, which was critically reviewed by SM, AS, MM, and AES, and further thoroughly revised by AES. All the authors reviewed the manuscript.

## Conflicts of interest

None of the authors have a conflicts of interest to disclose.

## Abbreviations

NBW: Natural biomass waste (—)
SFW: Synthetic fiber waste (—)
NWC: The natural waste-activated carbon (—)
SWC: The synthetic waste-activated carbon (—)
CCM: Composite carbon material (—)
BET: Brunauer–Emmett–Teller (—)
COD: Chemical oxygen demand (mg L^−1^)
BOD: Biochemical oxygen demand (mg L^−1^)
TSS: Total suspended solids (mg L^−1^)
MB_d_: Methylene blue dye (—)
AC: Activated carbon (—)
CAC: The commercially activated carbon (—)
*E*_r_
: Removal efficiency (%)
pH_c_: solution pH (—)
Ad_c_: adsorbent dosage (g)
*I*_c_
: Initial concentration (mg L^−1^)
*E*_c_
: Equilibrium concentration (mg L^−1^)
*T*_c_
: temperature (°C)
*t*_c_
: contact time (min)
DI water: Deionized water (—)
NaOH: Sodium hydroxide solution (—)
HCl: Hydrochloric acid solution (—)
SD: Standard deviation (—)
*C*_qe_
: The adsorption capacity at equilibrium (mg g^−1^)
OFAT: One-factor-at-a-time (—)
*C*_qmax_
: The maximum adsorption capacity (mg g^−1^)
*V*_d_
: Volume of the dye solution (L)
*m*_a_
: The mass of the adsorbent (g)
PFO_m_: Pseudo-first-order model (—)
PSO_m_: Pseudo-second-order model (—)
*C*_qt_
: The adsorption capacity at time (mg g^−1^)
*t*_m_
: Time (min)
*k*_1_
: The pseudo-first-order rate constant (min^−1^)
*k*_2_
: The pseudo-second-order rate constant (g mg^−1^min^−1^)
*b*_L_
: The Langmuir constant (L mg^−1^)
*K*_F_
: The Freundlich constant (mg g^−1^)(L mg^−1^)^1/*n*_*i*_^
*n*_*i*_
: Adsorption intensity (—)
*R* ^2^
: Correlation coefficient (—)
*T*_k_
: The absolute temperature (K)
*K*_c_
: The equilibrium constant (—)
*R*
: Universal gas constant (J mol^−1^ K^−1^)
Δ*G*_s_: The standard Gibbs free energy change (J mol^−1^)
Δ*H*_s_: The standard enthalpy change (J mol^−1^)
Δ*S*_s_: The entropy change (J mol^−1^ K^−1^)
SEM: Scanning electron microscopy (—)
TGA: Thermogravimetric analysis (—)
FTIR: Fourier transform infrared spectroscopy (—)
XRD: X-ray diffraction (—)
pH_zcp_: The point of zero charge (—)
pH_ci_: The initial pH_c_ (—)
pH_cf_: The final pH_c_ (—)
Zn: Zinc (—)
Cu: Copper (—)
Fe: Iron (—)
Cr: Chromium (—)
TDS: Total dissolved solids (mg L^−1^)
EC: Electrical conductivity (µS cm^−1^)
AAS: Atomic absorption spectrophotometry (—)
DAPZ: Distribution analysis of pore size (—)

## Data Availability

It is appropriate to request the datasets used and/or analyzed in this study from the corresponding author. The data collected and analyzed for this investigation are all contained in this published publication.
